# MultiMiTar: A Novel Multi Objective Optimization based miRNA-Target Prediction Method

**DOI:** 10.1371/journal.pone.0024583

**Published:** 2011-09-15

**Authors:** Ramkrishna Mitra, Sanghamitra Bandyopadhyay

**Affiliations:** Machine Intelligence Unit, Indian Statistical Institute, Kolkata, West Bengal, India; King Abdullah University of Science and Technology, Saudi Arabia

## Abstract

**Background:**

Machine learning based miRNA-target prediction algorithms often fail to obtain a balanced prediction accuracy in terms of both sensitivity and specificity due to lack of the gold standard of negative examples, miRNA-targeting site context specific relevant features and efficient feature selection process. Moreover, all the sequence, structure and machine learning based algorithms are unable to distribute the true positive predictions preferentially at the top of the ranked list; hence the algorithms become unreliable to the biologists. In addition, these algorithms fail to obtain considerable combination of precision and recall for the target transcripts that are translationally repressed at protein level.

**Methodology/Principal Finding:**

In the proposed article, we introduce an efficient miRNA-target prediction system MultiMiTar, a Support Vector Machine (SVM) based classifier integrated with a multiobjective metaheuristic based feature selection technique. The robust performance of the proposed method is mainly the result of using high quality negative examples and selection of biologically relevant miRNA-targeting site context specific features. The features are selected by using a novel feature selection technique AMOSA-SVM, that integrates the multi objective optimization technique Archived Multi-Objective Simulated Annealing (AMOSA) and SVM.

**Conclusions/Significance:**

MultiMiTar is found to achieve much higher Matthew’s correlation coefficient (MCC) of 0.583 and average class-wise accuracy (ACA) of 0.8 compared to the others target prediction methods for a completely independent test data set. The obtained MCC and ACA values of these algorithms range from −0.269 to 0.155 and 0.321 to 0.582, respectively. Moreover, it shows a more balanced result in terms of precision and sensitivity (recall) for the translationally repressed data set as compared to all the other existing methods. An important aspect is that the true positive predictions are distributed preferentially at the top of the ranked list that makes MultiMiTar reliable for the biologists. MultiMiTar is now available as an online tool at www.isical.ac.in/~bioinfo_miu/multimitar.htm. MultiMiTar software can be downloaded from www.isical.ac.in/~bioinfo_miu/multimitar-download.htm.

## Introduction

MicroRNAs (miRNAs) are tiny non-coding RNAs ∼ 22 nt of length that regulate their target genes at the post-transcriptional level either by degrading the target transcript or translationally repressing the corresponding protein product. In order to understand functional roles of the miRNAs and to asses their impact on target genes, accurate prediction of miRNA-target examples is necessary. Numerous target prediction algorithms have been proposed such as miRanda, [1], TargetScan [2], PicTar [3] and PITA [4] etc., including some machine learning based algorithms like NBmiRTar [5] and mirTarget2 [6] etc. However all of these suffered from either high false positive or false negative rates. In machine learning based miRNA-target prediction algorithms, the classifier needs to be trained with appropriate sets of positive and negative miRNA-target examples. A sufficient number of experimentally verified positive examples can be obtained from TarBase [7] and miRecords [8] database. However, these algorithms suffer from lack of gold standard of negative examples to build an effective classifier. This is because there are no assays demonstrating negative examples; these originate only from failed experiments of target validation. In the earlier machine learning approaches, randomly generated sequences were used as negative examples. However, these randomly generated negative examples may contain real cases by chance or may be unrealistically different from the positive set. As a result, artificially generated negative examples may produce a classifier that yields high cross-validation results, but poor performance on independent, real test data set. Systematic identification of more negative examples is therefore a critical issue for improving the accuracy of target prediction methods. A set of 289 tissue specific negative examples have been identified in [9] using a bioinformatic approach and proposed a target prediction method called TargetMiner. TargetMiner achieved the most balanced prediction accuracy in terms of sensitivity and specificity compared to the other methods and its robust performance is mainly because of the use of these high-quality of negative examples.

Although TargetMiner achieved the best result so far, it only used a naive feature selection technique. In general, selection of a subset of relevant informative features leads to a simpler model and often results in a better generalization performance. However, measuring the goodness of a selected subset of features using a single criterion may often become difficult. Therefore it may be more appropriate and natural to treat the problem of feature selection as one of multi-objective-optimization (MOO). In this paper our previously developed multi-objective simulated annealing based optimization method, Archived Multi-Objective Simulated Annealing (AMOSA) [10], is integrated with Support Vector Machine (SVM) in order to build AMOSA-SVM, a novel multi-objective based feature selection and classification tool. AMOSA is selected as the underlying optimization strategy as it has been shown to outperform several existing, popular MOO techniques. AMOSA-SVM extracts a set of informative, non-redundant features that enhance the predictive power of the proposed classifier MultiMiTar. MultiMiTar is found to achieve much higher sensitivity and specificity compared to 12 other existing target prediction methods for a completely independent test data set. Moreover, it achieves the balanced precision and recall for a large set of translationally repressed data experimented in [11] that has not been observed by the existing prediction methods.

For each test data point, MultiMiTar computes a prediction score. These scores have been taken into account in order to compute the ranks of the miRNAs that are targeting a single gene or for a set of genes that are targeted by a single miRNA. This, especially the ranking of the miRNAs, would be useful for the researchers because currently it is of prime interest to learn about the combinatorial interactions of the most favored miRNAs on a single target [12]. MultiMiTar is useful not only because of its robust performance but also of its ability to predict high confident interactions that are distributed preferentially towards the top of the ranked list. A detailed description of the proposed method and the data sets used to evaluate the performance of MultiMiTar are provided in the materials and methods section.

## Materials and Methods

This section first describes the data sets considered in this article. A set of biologically validated positive examples and a set of systematically identified tissue specific negative examples are considered as training examples. A set of 90 miRNA-targeting site context specific features have been extracted from the training data set. Among these 90 feature set, a subset of 39 more informative and relevant features have been extracted using our technique AMOSA-SVM, a novel integration of a multi-objective optimization (MOO) based feature selection tool AMOSA [10] and SVM classifier. Based on the selected subset of relevant features a classifier model has been built called MultiMiTar.

### Data sets

#### Experimentally verified positive and negative examples

A set of 289 biologically validated positive examples (see Table S1) and 289 systematically identified tissue specific negative examples (see Table S2) have been extracted from [9] as training data sets in order to build the classifier model. A completely independent biologically validated test data set is considered. The data set consists of 187 positive and 57 negative examples among which randomly selected 10 positive and equal number of negative examples are separated to generate a small independent validation data set (see Table S3). This data set is used for finding the optimal parameters of the classifier SVM. Rest of the data set (Table S4 and Table S5) is used to assess the prediction performance of the proposed MultiMiTar compared to the 12 other target prediction methods.

#### miRNA and 3′ UTR sequence data set

All the available human mature miRNA sequences are collected from miRBase database (http://microrna.sanger.ac.uk/sequences). Human 3′ UTRs are extracted from the University of California, Santra Cruz (UCSC) Genome bioinformatics site (http://genome.ucsc.edu).

#### pSILAC data set

From the pSILAC (pulsed stable isotope labelling with amino acids in cell culture) data [11], 15,806 examples have been extracted in [13]. In this data set, there are 2,406 mRNAs that are downregulated more strongly than −0.2 log-fold change at protein level. In the present article, these are considered as targets and the remaining 13,400 examples are considered as non-targets as described in [13].

### Extraction of Features

**Figure 1:**
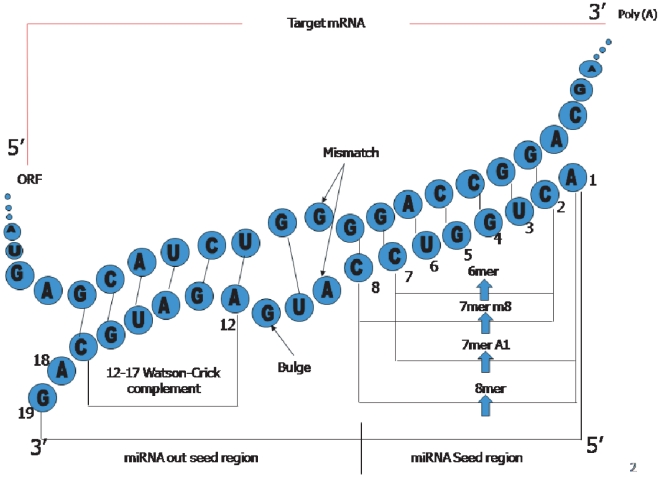
Different miRNA-mRNA seed-site interaction patterns (6mer, 7mer-A1, 7mer-m8 and 8mer). Watson-Crick complimentary regions can be obtained at miRNA seed and out-seed part.

We have generated a set of 90 miRNA-targeting site context specific features. These are described here for the convenience of the reader. MiRNA sequence is divided into seed (position 1 to 8) and out-seed regions (remaining part). Seed matching site is categorized into 6mer/7mer-A1/7mer-m8/8mer. These are recognized as functional because target mRNAs with one or more of these seed matching sites are preferentially downregulated [14]. A perfect seed matching site of length 6 including one optional GU wobble pair (miRNA seed region 2–7/3–8) is considered as 6mer. MultiMiTar first search the 6mer seed complementary sites in the 3′UTR of mRNA. A single GU wobble pair is considered, if present in the seed matching site. A 6mer seed matching site (miRNA seed region 2–7) including another complementary pair at position 8 of miRNA seed is referred to as 7mer-m8. In case of category 7mer-A1, position 1 of miRNA is aligned with ‘A’ of target 3′UTR including one 6mer seed matching site (miRNA seed region 2–7). Presence of both the 7mer-m8 and 7mer-A1 can be categorized into 8mer seed matching site (for details see Figure 1).

For miRNA-target interaction, an additional Watson-Crick base pairing may be present at miRNA positions 13–16 and that can be extended to position 12 and 17 of miRNA out-seed part. If so, then the corresponding feature value is set to 1, otherwise 0 is considered (see Figure 1). We have also measured whether the seed-site is preferentially residing within a locally AU rich region or not. Functional sites are highly enriched for A and U content relative to the non functional sites; local AU contents impact not only mRNA destabilization but also protein expression. For doing this we have considered the composition of bases from the upstream and downstream flanking region (30 nt each) of the seed site. We set the feature value to 1 if in this region the AU content is ≥ 60%, otherwise this is set to 0. All these biologically explained features are grouped into category 1. Beside this, other categories of features are (2) Frequency of Single nucleotide in seed matching site (3) Frequency of Single nucleotide in seed matching out site (4) Frequency of di-nucleotides in seed matching site (5) Frequency of di-nucleotides in seed matching out site (6) miRNA-mRNA base interaction features in seed region and (7) Two consecutive miRNA-mRNA base interaction features in seed region (bi-di-nucleotide base pairing) (see Table S6).

In feature category 2 and 4, frequency of single nucleotides (A, C, U, G) and dinucleotides is computed by considering the seed matching site region. Unlike to most of the existing target prediction algorithms, MultiMiTar is not restricted for considering target information only from seed-site interaction regions. Rather, in feature category 3 and 5 we have considered immediately flanking regions of seed-interaction site. This region is important because the sequence surrounding the target site is assumed to take an effective part for the accessibility of target site by the miRNA [4]. Hence, it is expected that these regions provide discriminating and informative features for positive and negative examples. In this regard length of the flanking regions is an important factor. We have considered 30 nt upstream and 30 nt downstream sequences from the seed matching site as the effective region. This is biologically a valid region (60nt+Nmer site) as there is a less probability of intra mRNA base pairing interactions between bases that are separated by more than 70 nucleotides [4].

In category 6, frequency of 6 types of base pair interaction viz., A:U, U:A, G:C, C:G, G:U and U:G at seed matching site is possible. In the proposed approach a single GU wobble pair is considered at seed-site interaction region. We observe that GU pairing at seed-site region may have a partial influentially role in identification of potential positive examples. A detailed description is provided in section discussion. In category 7, we have extracted the frequency of two consecutive base pairing. For example, frequency of the occurrence of A:U base pairing immediately after C:G.

### Classifier model building based on SVM

The SVM is a supervised learning algorithm [15] [16] that can learn the classifier by transforming the input data into another higher dimensional feature space where it is easy to compute an accurate classification. In this study, a training vector corresponds to the miRNA-targeting site context specific features. Given 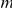 training vectors 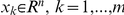 and a vector of labels 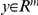 such that 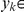 {1,−1} (+1 for miRNA-target mRNA, -1 for miRNA-non target mRNA), the SVM in training learns a hyperplane (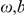), optimally separating the items of the two classes, defined as:
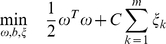
(1)


subject to
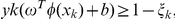


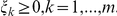



The function 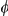 maps the training data to a higher dimensional space and 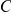 is a penalty parameter on the training error. With a learned hyperplane (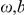), a query vector 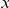 (miRNA-targeting site context specific feature vector) can be classified based on the decision value 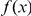 or the SVM score:
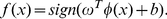
(2)


We need a kernel function 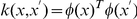 for mapping the data to a higher dimensional space. In this regard a radial basis function (RBF) kernel is used,
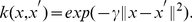
(3)


The query vector 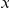 is compared with the feature vector of each of the training instance using the radial basis function in order to calculate the decision value 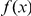. From the sign of 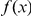 a decision is made whether it is from class target or non-target. A decision value which is the distance between each input vector and a decision plane can be used to evaluate the reliability of the prediction [17]. In general, the prediction with a higher decision value is considered as more reliable [16]. The obtained decision value has been considered and reported as the MultiMiTar prediction score.

Finding of an optimal hyperplane depends on the selection of the two parameters 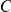 and 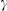. The parameter 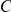 is used to control the tradeoff between the training error and the margin, and the parameter 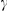 controls the width of the RBF kernel. We iteratively build the classifier model for the training data set with different combinations of 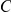 and 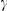 values and measure the performance of the classifier based on a small independent validation data set (see Table S3). The optimal parameters are chosen from the classifier model that shows the best prediction accuracy on this small data set.

### Feature selection algorithm AMOSA-SVM: A novel integration of AMOSA and SVM

AMOSA is an MOO based generalized version of simulated annealing (SA) [10]. In the proposed article AMOSA has been integrated with SVM in order to build a feature selection and classification technique AMOSA-SVM. SA is a popular search technique that can solve single objective based optimization problems. However, its utility has been limited in solving the MOO problem because of its point based search nature. AMOSA can efficiently overcome the limitation of SA to solve the MOO problem. The MOO can be stated as follows [18]: determine the vectors 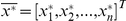 of decision variables that simultaneously optimize the N objective values 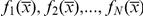, while satisfying the constraints, if any. In the MO maximization framework a solution 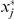 is defined to be dominated by another solution 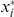 if 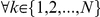, 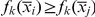. Among the set of solutions S, a subset is considered as non-dominated if none of the solutions in it is dominated by any member of the set S. In general, an MOO algorithm defines a set of solutions that are not dominated by any solution encountered by it.

In the AMOSA algorithm the non-dominated solutions are stored in an archive. The archive maintains two limits viz., a hard limit denoted by 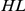 and a soft limit denoted by 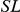. At the very beginning, the algorithm considers 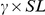 (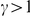) number of initial solutions each of which represents a state in the search space. The multiple objective functions are computed. For a predefined number of iterations, each of these solutions is refined by using a simple hill climbing technique. Here a current solution is perturbed to generate a new solution which is accepted if it dominates the previous one. Finally, the non-dominated solutions are stored in the archive. A single linkage clustering scheme is used when the archive size exceeds 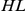. From each cluster the member whose average distance to the other members is the minimum, is considered as the representative member of the cluster. This completes the archive initialization process. Now from the archive one point is selected and considered as *current-pt* or the initial solution at an initial temperature *Tmax*. The *current-pt* is perturbed and a new point called *new-pt* is generated. The objective functions of the *new-pt* are computed. In order to accept or reject the new solution, AMOSA uses the concept of amount of domination to measure the acceptance probability of the new solution. For the two solutions 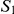 and 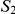 the amount of domination is computed as,
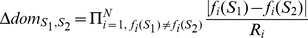
(4)


where 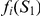 and 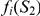 are the 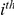 objective values of the two solutions, 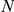 =  number of objectives and 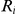  =  range of the 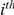 objective. Domination status determines different conclusions such as accept the (a) *new-pt* (b) *current-pt* or (c) an existing solution from the archive [10]. After storing the accepted solution in the archive, AMOSA checks whether the archive size exceeds 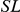. In this case, single linkage clustering is applied to reduce its size to 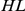. For each temperature the process is repeated for a predefined number of iterations. The process is annealed with a cooling rate of 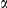 (here 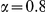) till the minimum temperature *Tmin* is attained. Then the process is terminated with a set of non-dominated solutions stored in the archive.

Selection of relevant feature set is crucial to enhance the predictive power of any classifier. In the proposed AMOSA-SVM, a state of AMOSA denotes the features that are selected to build the SVM classifier model. The selected and discarded features are denoted by 1’s and 0’s, respectively. Hence, a string of 1’s and 0’s of length 90 indicates the features that are to be used (the 1’s) for building the SVM. In order to evaluate the performance of the classifier, three objectives such as sensitivity (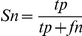), specificity (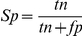) and Matthew’s correlation coefficient (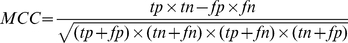) are computed. These objective values are used to accept and store the relevant solutions in the archive. The chosen objectives should be equally important. Here, 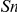 and 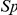 control false negatives and false positives, respectively and 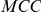 balances the classification results. For each data point the objective functions are computed based on 5-fold cross validation using radial basis function (RBF) kernel. Among a set of non-dominated solutions stored in the archive, we select the one that achieves the highest accuracy based on perfectly balanced training data. The selected solution consists of 39 relevant features (see Table 1) that have been used to build the SVM classifier model using publicly available tool Libsvm [19].

**Table 1:** miRNA-targeting site context specific relevant features used in MultiMiTar.

Feature number	Feature name	Common features
	Category 1	
6	Number of additional Watson-Crick pairing associated with effective seven mer m8	*
	Frequency of Single nucleotide in seed matching out site (Category 3)	
19	G’s frequency in effective seed matching out site	
	Frequency of Di-nucleotides frequency in seed matching site (Category 4)	
22	AU’s frequency in effective seed matching site	
24	AC’s frequency in effective seed matching site	
25	UA’s frequency in effective seed matching site	
26	UU’s frequency in effective seed matching site	*
28	UC’s frequency in effective seed matching site	*
30	GU’s frequency in effective seed matching site	*
32	GC’s frequency in effective seed matching site	
35	CG’s frequency in effective seed matching site	*
36	CC’s frequency in effective seed matching site	*
	Frequency of Di-nucleotides in seed matching out site (Category 5)	
38	AU’s frequency in effective seed matching out site	
39	AG’s frequency in effective seed matching out site	*
40	AC’s frequency in effective seed matching out site	*
42	UU’s frequency in effective seed matching out site	*
44	UC’s frequency in effective seed matching out site	*
45	GA’s frequency in effective seed matching out site	
47	GG’s frequency in effective seed matching out site	
48	GC’s frequency in effective seed matching out site	
	miRNA-mRNA base interaction features in seed region (Category 6)	
53	Frequency of AU base pair	*
54	Frequency of UA base pair	
56	Frequency of GC base pair	*
57	Frequency of GU base pair	*
58	Frequency of CG base pair	*
	Two consecutive miRNA-mRNA base interaction features in seed region (Bi-Di-nucleotide base pairing) (Category 7)	
59	Frequency of AU-AU	*
62	Frequency of AU-CG	*
64	Frequency of AU-UG	*
65	Frequency of UA-AU	
67	Frequency of UA-GC	
68	Frequency of UA-CG	*
69	Frequency of UA-GU	
70	Frequency of UA-UG	*
73	Frequency of GC-GC	
74	Frequency of GC-CG	
78	Frequency of CG-UA	*
79	Frequency of CG-GC	*
83	Frequency of GU-AU	
84	Frequency of GU-UA	*
86	Frequency of GU-CG	

The features are selected by using novel feature selection algorithm AMOSA-SVM. Category-wise list of common features selected by at least 90% non-dominated solutions in the archive are denoted by ‘*’.

## Results

In this section we report the comparative performance of MultiMiTar vis-a-vis several existing methods, for a completely independent test data set. In a part of the experiment, a detailed analysis of the feature set selected by MultiMiTar is conducted.

### Performance on completely independent test data

**Figure 2:**
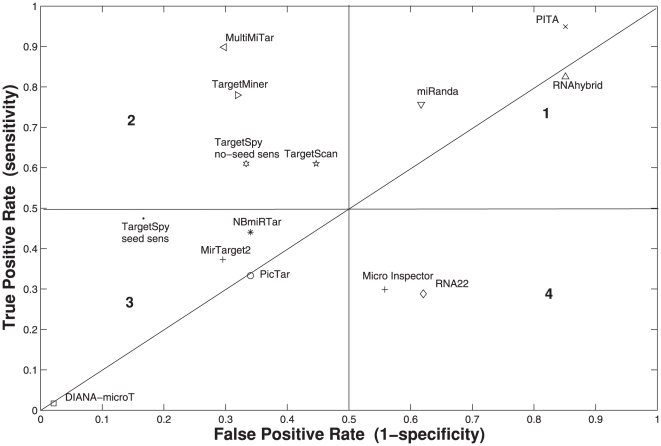
Scatter plot of the True positive rate versus the False positive rate for different algorithms. The plot is based on the independent test data set.

Figure 2 shows the plot for the true positive rate versus the false positive rate on the completely independent test data set. In this regard, thirteen target prediction algorithms including the recently published target prediction method TargetSpy [20] have been considered. TargetSpy provides prediction results for no seed match requirement (*TargetSpy no-seed sens*/*TargetSpy no-seed spec*) and seed match requirement (*TargetSpy seed sens*/*TargetSpy seed spec*) where sens and spec correspond to two threshold scores as mentioned in [20]. The plot compares the balance between sensitivity and specificity of the proposed method MultiMiTar with other existing methods. The plot area is divided into four quadrants marked 1 to 4 for the convenience of the readers. The diagonal line (0,0) – (1,1) denotes an algorithm that produces equal of true positive and false positive rates, i.e. a totally random method without any predictive power. The four quadrants denote algorithm that achieve the following: (1) higher sensitivity but lower specificity, (2) higher sensitivity and higher specificity, (3) lower sensitivity but higher specificity and (4) lower sensitivity and lower specificity. Evidently, the algorithms in quadrant 2, far away from the diagonal line are better performers. According to Figure 2, MultiMiTar is plotted further away from TargetMiner, TargetScan and *TargetSpy no-seed sens* (and *TargetSpy no-seed spec*) and closer to the optimal performance point i.e. (1,1). *TargetSpy no-seed spec* obtains similar results as that of *TargetSpy no-seed sens*, hence this has not been plotted in the Figure 2.

To evaluate the prediction sensitivity and specificity of MultiMiTar compared to TargetMiner, the area under the curve (AUC) has been computed. It is found that TargetMiner and MultiMiTar have AUCs of 0.7085 and 0.7464, respectively, clearly depicts the fact that the sophisticated AMOSA based feature selection in MultiMiTar is primarily responsible for its improved performance. A detailed comparative results in terms of MCC and ACA are shown in Table 2. As is evident, MultiMiTar provides the best values of MCC (0.583) and average class-wise accuracy (ACA) (0.8) compared to the other existing algorithms including TargetMiner (MCC =  0.403 and ACA =  0.73), TargetScan (MCC =  0.135 and ACA =  0.582) and *TargetSpy no-seed sens* (MCC =  0.209 and ACA =  0.56) that are placed at quadrant 2.

**Table 2:** Performance of MultiMiTar and existing target prediction methods on independent test data set.

Method	MCC	ACA
MultiMiTar	0.583	0.800
TargetMiner	0.403	0.730
PITA	0.155	0.549
TargetScan	0.135	0.582
miRanda	0.128	0.570
NBmiRTar	0.083	0.550
MirTarget2	0.052	0.495
PicTar	−0.006	0.496
DIANA MicroT 3.0	−0.013	0.498
RNAhybrid	−0.029	0.487
MicroInspector	−0.216	0.378
RNA22	−0.269	0.321
TargetSpy no-seed sens	0.209	0.560
TargetSpy no-seed spec	0.209	0.560
TargetSpy seed sens	0.234	0.557
TargetSpy seed spec	0.234	0.557

### Feature analysis

As already mentioned, while TargetMiner uses a simple way to select features (using the F-score), MultiMiTar uses a sophisticated MOO based approach. The proportion of the features selected in the two techniques is not the same. The ratio of the number of selected features to the total number of features in each category, is referred to as the feature selection ratio (FSR). For each category the obtained FSRs for TargetMiner and MultiMiTar have been shown in Table 3. We can see that the obtained FSR for TargetMiner and MultiMiTar are quite different. For example, for the features that are extracted from category 1, the FSR for TargetMiner is quite high at 41.67%, while that of MultiMiTar is very low at 8.33%. For all the features of this category the obtained correlation coefficient between biologically validated positive and negative examples is very high (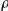 =  0.964, Pearson’s product-moment correlation, see column 3 of Table 3) indicating that possibly this is not a good discriminating feature set. Hence considering lesser number of features from this category as in MultiMiTar appears to be proper. Similarly, considering more number of features would be useful if the feature has a poor correlation between positive and negative data set. For example, for the categories 4 and 7, the correlation coefficients are comparatively poor (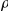 =  0.734 and 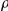 =  0.784) where MultiMiTar has higher FSRs of 56.25% and 46.87%, respectively, compared to TargetMiner which has 18.75% and 12.5%, respectively.

**Table 3:** Category-wise feature selection ratio for TargetMiner and MultiMiTar.

Feature	Total	Corr-coeff	TargetMiner		MultiMiTar		Common feat. in archive	
category	Feat.		No of Feat.	Ratio(%)	No of Feat.	Ratio(%)	No of Feat.	Ratio(%)
1	12	0.964	5	41.67	1	8.33	1	8.33
2	4	0.90	2	50	0	0	0	0
3	4	0.984	2	50	1	25	0	0
4	16	0.734	3	18.75	9	56.25	5	31.25
5	16	0.976	11	68.75	8	50	4	25
6	6	0.865	3	50	5	83.33	4	66.67
7	32	0.784	4	12.5	15	46.87	8	25
Total	90		30	33.33	39	43.33	22	24.44

In the proposed work, AMOSA-SVM stores a set of non-dominated solutions in the archive each representing a subset of the relevant features. The feature set encoded in each solution need not to be the same; however it is interesting to analyze the features that are common to at least 90% of the solutions (referred to as common feature set). These features are mentioned in Table 1. The last column of Table 3 shows FSR rate of the common features grouped into different categories. In order to study the importance of the different categories of features, we do the following. For each feature category, the features that in the common feature set are removed from the 39 features and the performance of the classifier is measured based on the completely independent test data set mentioned above. Removing common features from category 5 or 6 result in lower performance (AUC = 0.7262 and AUC = 0.7241, respectively) of the classifier compared to that for 39 features (AUC = 0.7464). The other categories of features are found to produce very small change of performance of the classifier (AUC of category 1 = 0.7358, category 4 = 0.7353 and category 7 = 0.7364). This clearly demonstrates that the common features of category 5 and 6 are relevant and these increase the predictive power of the classifier.

### Detection of downregulated proteins based on pSILAC data

**Figure 3:**
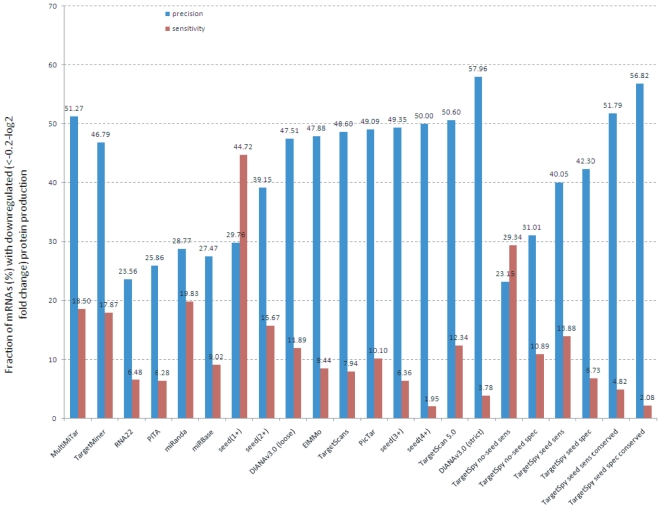
Performance comparison of several miRNA target prediction algorithms on the Psilac data. Proteins with log2-fold change <−0.2 are considered as target.

Due to the over/under-expression of a transfected miRNA into HeLa cells, changes of protein levels had been measured in [11] in order to assess the endogenous regulation of mRNA translation by miRNAs. In [11] those mRNAs that were downregulated more strongly than −0.1 log2-fold change at protein level by any of the five miRNAs viz., hsa-let-7b, hsa-miR-1, hsa-miR-16, hsa-miR-30a and hsa-miR-155 were considered to be the targets. In [13], proteins that were downregulated more strongly than −0.2 log2 fold change by any of the five miRNAs were taken as targets. This stricter definition in [13] than in the original paper makes the data more reliable towards assessing the prediction accuracy of target prediction algorithms. In total 15,806 interactions are observed in [13] among which a total of 2,406 interactions were found where mRNAs are downregulated more strongly than −0.2 log2-fold change at protein level. Hence are considered as targets in the present paper (see Dataset S1). The remaining 13400 examples that have log2 fold change of ≥−0.2 are considered as non-targets (see Dataset S2). According to [13], only two algorithms viz., TargetScan 5.0 [2] and DIANAv3.0(strict) [21] obtained a precision (the fraction of the predicted targets that are downregulated) of 50%. However, these algorithms fail to obtain a good sensitivity (recall) for the mRNAs that are downregulated at the protein level mentioned above. The obtained sensitivities for these two algorithms are 12.34% and 3.78%, respectively (see Figure 3). For the rest of the algorithms, miRanda obtained a comparatively better recall of 19.83%, however it is suffered from lower precision of ∼28.77 only. In [13], it has been shown that nearly half of the downregulated genes consist of at least one miRNA seed interaction site. As can be shown in Figure 3 a simple seed measure (seed (1+)), therefore, provides a good recall of 44.72% with the expense of lower precision (29.76). Here, additionally we have measured the precision and recall for MultiMiTar, TargetMiner and TargetSpy algorithms. For this data set, we have extracted six types of prediction results provided by TargetSpy. These are *TargetSpy no-seed sens*, *TargetSpy no-seed spec*, *TargetSpy seed sens*, *TargetSpy seed spec*, *TargetSpy seed sens* for conserved data and *TargetSpy seed spec* for conserved data where sens and spec correspond to two threshold value mentioned earlier. As can be seen from Figure 3, *TargetSpy seed sens* for conserved data and *TargetSpy seed spec* for conserved data obtained a precision of 51.79% and 56.82%, respectively. However, again these two suffered from a very low recall of 4.82% and 2.08%, respectively. On the otherhand, MultiMiTar obtains the precision of 51.27% with a good recall of 18.50% compared to the others (see Figure 3) including TargetMiner (precision =  46.79% and recall =  17.87%). For this experiment, we have considered conserved targets for a phastcons cutoff value of 0.57 as almost all the other target prediction methods consider conservation criteria. A detailed prediction results for the downregulated and upregulated data set are provided in Dataset S3 and Dataset S4 respectively. As can be seen from Figure 3, among 12 predictors that are obtaining precision of ≥ 45%, only MultiMiTar and TargetMiner obtain recall of >15% (18.5% and 17.87%, respectively). Among the rest of the methods DIANAv3.0 (loose) and TargetScan5.0 obtain a recall of 11.89% and 12.34%, respectively. Rest of the algorithms obtain recall values from 1.95% to 10.10%. This clearly demonstrates that MultiMiTar achieves a balanced result in terms of precision and recall, while the existing target prediction algorithms suffer either from poor precision or recall for the pSILAC validated translationally repressed data set.

### Predicting true positive examples: A ranking analysis

In the proposed MultiMiTar prediction result, two types of rankings have been introduced viz., (i) ranking of the miRNAs from their combinatorial interactions on a single target and (ii) ranking of the mRNAs targeted by a single miRNA. Specifically ranking of the miRNAs that are targeting a single gene of interest is in high demand. Investigators are interested to obtain only reliable, high confident miRNAs that are targeting a gene of interest involved in specific disease such as cancer. Therefore a common approach is to consider the top ranked miRNA molecules and verify their targeting potentiality experimentally. In the proposed work a comparative study has been conducted to know whether the existing target prediction algorithms can efficiently detect biologically validated combinatorial interactions of several miRNAs on a particular target transcript as top ranked examples. Recently in [12], a high throughput luciferase reporter screen demonstrated that p21Cip1/Waf1 gene can be directly targeted by a large set of 28 miRNA molecules. p21Cip1/Waf1, also known as Cyclin-dependent kinase inhibitor 1A (CDKN1A) acts as a master effector molecule of multiple tumor suppressor pathways. Till date this is the only gene that has been verified experimentally to be regulated by such a large number of miRNAs. On the basis of this combinatorial interaction data set we measure the sensitivity and ranks of the miRNAs that have been predicted to target CDKN1A by using MultiMiTar and existing target prediction algorithms.

The prediction accuracies obtain by the existing algorithms have been provided in Table S7. As is evident from the table, MultiMiTar obtains a sensitivity (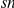) of 67.86% which is much better compare to the three most popular methods viz., miRanda (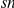 = 60.71%), PicTar (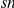  =  21.43%) and TargetScan (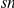  =  28.57%). Some other methods viz., microInspector (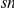  =  25%) [22], Diana-microT 3.0 (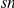  =  39.28%) and the two machine learning based methods MirTarget2 (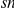  =  28.57%) and NBmiRTar (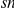  =  46.43%), have poor sensitivity. In contrast PITA and RNAhybrid appear to recognize almost all the 28 miRNAs correctly. However this is quite misleading since these two tend to predict all the examples as positive as is evident from Figure 2.

**Figure 4:**
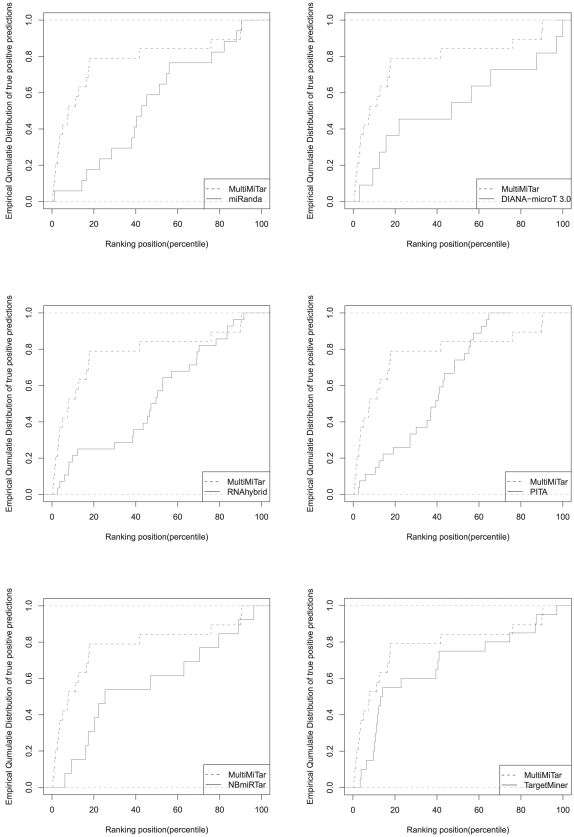
Distribution of the predictions of MultiMiTar and other algorithms in recognizing biologically validated miRNA-CDKN1A interactions. The plots show that MultiMiTar obtains the most preferential distribution that tends to be shifted towards the top 20th percentile compared to the other algorithms.

We now consider MultiMiTar and the six most sensitive target prediction methods including TargetMiner. For each algorithm we observe whether the true positive predictions (the number of correctly predicted positive examples) are uniformly distributed along the entire ranked list or distributed preferentially at the top of the ranked list. We found that, 78.95% of the total true positive predictions fall within 20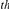 percentile of MultiMiTar ranked list; this is very high compared to the other algorithms used in this study. For example, only 17.65%, 30.77%, 25.93%, 25%, 36.36% and 55% true positive predictions lie within the 20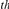 percentile of the ranked list of miRanda, NBmiRTar, PITA, RNAhybrid, DIANA-microT 3.0 and TargetMiner, respectively (see Figure 4). This clearly elucidates the fact that the ranking provided by MultiMiTar is superior to the ranking provided by the existing target prediction algorithms including TargetMiner. The obtained P-values (Wilcoxon rank sum test) between MultiMiTar and the rest of the algorithms show the statistically significant superiority of the former algorithm over the others (see column 2 of Table 4). On the other hand, except for the case between TargetMiner and miRanda (*P*-value 3.72×10^−02^, Wilcoxon rank sum test) there is no statistically significant difference between any two ranked lists among the rest of the algorithms (see column 3–7 of Table 4). This clearly depicts the fact that excluding MultiMiTar, all the prediction methods are less reliable and are unable to keep true positive predictions at the top of the ranked list.

**Table 4:** Pairwise comparisons between different ranked lists distributed preferentially (MultiMiTar) or uniformly (rest of the algorithms).

	MultiMiTar	miRanda	NBmiRTar	PITA	RNAhybrid	DIANA-microT 3.0
miRanda	1.73×10^−03^	–	–	–	–	–
NBmiRTar	4.91×10^−03^	0.34	–	–	–	–
PITA	3.45×10^−03^	0.15	0.34	–	–	–
RNAhybrid	1.57×10^−03^	0.43	0.45	0.06	–	–
DIANA-microT 3.0	1.32×10^−02^	0.50	0.50	0.23	0.42	–
TargetMiner	4.2×10^−02^	3.72×10^−02^	0.12	0.15	0.10	0.13

P-values are obtained by wilcoxon rank sum test.

### Comparative study of MultiMiTar and TargetMiner: A statistical analysis

Sophisticated AMOSA based feature selection in MultiMiTar is primarily responsible for its improved performance over TargetMiner. For different data sets, it is important to measure whether such improved performances are statistically significant. Moreover, it would be meaningful to observe whether for the other data sets also, ranking of the miRNAs obtained by the proposed method is significantly better compared to TargetMiner, especially because the proposed method is motivated by TargetMiner.

### Comparison based on *Sn*, *Sp*, *MCC* and *Precision*

We have reconsidered the two data sets viz., independent test data and the pSILAC data [11] (described in section Materials and Methods). For the independent test data, a set of 100 miRNA-mRNA pairs (both positive and negative examples) are extracted randomly. Based on this, the prediction has been carried out using MultiMiTar as well as TargetMiner. The classification results are measured in terms of *Sn*, *Sp*, *MCC* and *Precision*. The process is repeated 100 times and results are stored. Based on the obtained results, a non-parametric Wilcoxon rank sum test, at 0.05 level of significance, is carried out to measure whether the proposed method obtains a statistically significant improved performance in terms of *Sn*, *Sp*, *MCC* and *Precision* over TargetMiner. The P-values obtained were *Sn*  =  <2.2×10^−16^, *Sp*  =  0.1152, *MCC*  =  <2.2×10^−16^ and *Precision*  =  2.453×10^−5^. The results show that except for *Sp*, for the other measures the proposed method achieves a statistically significant superior performance compared to that of TargetMiner. In terms of *Sp*, an improved performance is observed for MultiMiTar (see Fig. 2), but this is not statistically significant. This is because of the small number of available negative examples used in the experiment.

For the conserved pSILAC data, 10% of the miRNA-mRNA pairs (both positive and negative examples) are randomly selected and similar experimentation, as described above, is carried out. Again, the P-values for *Sn*  =  1.043×10^−02^, *Sp*  =  7.327×10^−14^, *MCC*  =  2.202×10^−11^ and *Precision*  =  4.024×10^−8^ clearly elucidate the fact that the proposed method provides an improved classification result which is statistically significant compared to TargetMiner. Note that, for this data set where a large set of negative examples are available, in terms of the specificity, a remarkable improvement in prediction is observed by the proposed method compared to TargetMiner.

### Comparison based on ranking

**Figure 5:**
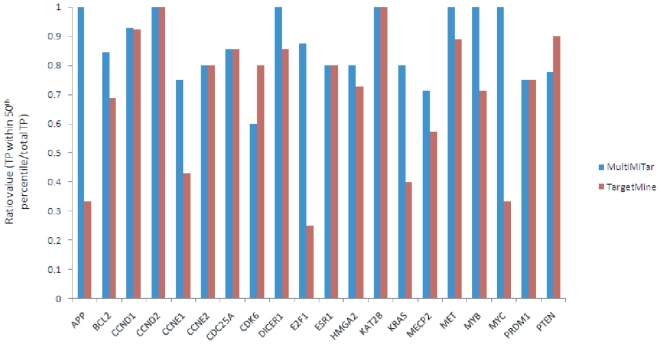
Comparison between MultiMiTar and TargetMiner based on ranking results for true positive examples.

Here, we have considered more data sets to check whether the proposed method obtains consistently better rankings compared to that of TargetMiner, the second best ranking provider (see Figure 4 and Table 4). In this regard 20 mRNAs, each of which is targeted by 6 or more miRNAs, are extracted from miRTarBase [23], a recently published database. These 20 mRNAs constitute 20 biologically validated data sets. For each mRNA, the proposed method MultiMiTar predicts a list of miRNAs. For each data set, we count how many true positive examples fall within 50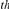 percentile of MultiMiTar predicted ranked list and divide it by the total true positive predictions to yield the ratio value. Similar tasks have been carried out by TargetMiner. A vector of such ratio values is generated by each of the algorithms (see Figure 5). The higher the ratio value is, the more the chance that the prediction algorithm is getting superior compared to the other one. As can be seen from Figure 5 that out of the 20 mRNAs, the proposed method obtains higher, same and lower ratio values for the 12, 6 and 2 mRNAs, respectively, compared to that of TargetMiner. A t-test has been carried out based on the list of 20 ratio values provided by each of the two algorithms. The result (*P*-value 4.72×10^−03^) clearly demonstrates the fact that the proposed method provides an improved ranking result which is statistically significant.

## Discussion

This paper describes MultiMiTar, a novel integration of MOO-based feature selection and classification for miRNA target prediction. MultiMiTar obtains the best MCC of 0.583 and ACA of 0.8 compared to the existing 12 target prediction methods including TargetMiner (MCC =  0.403 and ACA =  0.73). Beside MultiMiTar and TargetMiner, rest of the 11 target prediction methods obtain the MCC and ACA ranges from −0.269 to 0.155 and 0.321 to 0.582, respectively. MultiMiTar seems to be the best target prediction algorithm so far compared to the others. However, before drawing any conclusion in favor of the proposed algorithm, it should be tested on different data sets that have distinct characteristics from each other. In this regard we have considered a pooled dataset of mRNA cleavage examples extracted from TarBase (Papadopoulos et al., 2009), experimentally verified interactions between RAS protein and hsa-let-7 family miRNAs ([24] and [25]), a data set consisting of combinatorial interactions between p21Cip1/Waf1 gene and 28 human miRNAs experimented in [12], and the largest set of translationally repressed miRNA-target examples obtained from [11].

In [9], performance comparisons among several target prediction algorithms had been carried out based on a pooled data set of mRNA cleavage examples. We have considered this data set and measured the performance of MultiMiTar compared to the other existing popular target prediction algorithms which were mentioned in [9]. For this data set, the sensitivities of MultiMiTar, TargetMiner, TargetScan, PicTar, miRanda, MirTarget2 and NBmiRTar are 0.868, 0.816, 0.790, 0.684, 0.658, 0.658 and 0.526, respectively. MultiMiTar has already shown a superior performance for the translationally repressed (PSILAC data) data set. Here, it obtains the best sensitivity for the cleavage data set as well. These results clearly depict the fact that MultiMiTar is able to predict both types of miRNA regulation and dominates the other existing target prediction methods.

The influence of GU pairing at miRNA-target seed interaction site has been addressed in [25]. According to [25], most of the target prediction algorithms fail to obtain a good prediction accuracy as they do not tolerate non-Watson-Crick seed pairing (e.g. TargetScan is unable to predict a single seed match in let-7 miRNAs-KRAS interaction). However, we observe that GU pairing at seed-site region may have a partial influentially role rather a full control. In this regard we have considered KRAS and all the let-7 miRNAs and measured the performance of MultiMiTar and all the major target prediction algorithms viz., miRanda, TargetScan and PicTar. All the major prediction algorithms have failed to predict a single positive example whereas MultiMiTar obtained the optimal prediction accuracy (100%). Although MultiMiTar considers a single GU pairing at seed matching site, we further investigate if the good prediction accuracy is due to the inclusion of this feature. So we build MultiMiTar classifier model without considering single GU pairing at the seed matching site and measure its performance on this data set again. Still an optimal prediction accuracy (100%) has been observed. However, now it detects a smaller number of seed matching sites with lower decision values compared to the original MultiMiTar (see Table S8a and Table S8b). This clearly depicts the fact that for this data set, considering GU pairing at the seed matching site plays a partial role for identifying true positive examples.

Based on the data set experimented in [12] it has been observed that MultiMiTar is so far the best algorithm that can predict high confident interactions that are distributed preferentially towards the top of the ranked list (as discussed in previous section, Figure 4 and Table 4). This is an useful experiment through which researchers can rely on the proposed algorithm and may use its top ranked predicted target set for their desired work.

For the translationally repressed target set [11] of 5 miRNAs that are downregulated more strongly than −0.2 log2-fold change at protein level, MultiMiTar provides the most balanced result in terms of precision and recall as compared to the others. This data set provides thousands of negative examples and hence it is possible to measure the specificity of different target prediction algorithms. This experiment is useful because in reality there are a lot more of negative examples than positive examples. Hence, the motivation of target prediction algorithms is to predict only those small number of true targets. Through pSILAC data set, it has already been observed that the existing target prediction algorithms that provide a high sensitivity for the biologically validated positive examples suffer from low specificity. These algorithms would be unreliable to the biologists because of the very high false positive prediction rate. On the other hand, those algorithms that provide a high specificity would fail to detect many true targets or have very high false negative prediction rate. Only a few target prediction algorithms, like MultiMiTar, exist that provide a balanced prediction rate. Using MultiMiTar we have searched for human genome-wide potential conserved targets as in miRanda, TargetScan and PicTar, etc (we used phastcons cutoff value of 0.57). The average number of predicted targets for a miRNA is moderate (1079.45). Moreover, the targets are associated with a score (decision value), making it possible for the users to select only a few top ranked targets for future study. The predicted targets are available in www.isical.ac.in/~bioinfo_miu/multimitar-genomewide-prediction.zip. We have extracted statistics from [8] and observed that those algorithms that provide a high sensitivity for biologically validated positive examples also provide a very high false positive rate. For example, RNAhybrid, PITA, and miRanda obtained high sensitivity for the different experimental data sets and the average number of predicted targets for these algorithms are 10958, 3956 and 3005, respectively. On the otherhand, it has been observed that, although average prediction rates for PicTar, MirTarget2 and TargetScan are comparatively low (200, 255.3 and 685.9, respectively), these suffer from lower sensitivity (or provide a high false negative rate). Moreover, as observed from Figure 3, the specificities obtained by these algorithms are also lower compared to MultiMiTar. A sophisticated and robust target prediction algorithm should provide a balanced sensitivity and specificity over different types of data sets used in the proposed article. These data sets clearly depict the fact that MultiMiTar is the most reliable and robust algorithm so far among the existing popular methods.

## Supporting Information

Table S1 **List of 289 positive training examples with references.**
(DOC)

Table S2 **List of 289 tissue specific negative training examples.**
(DOC)

Table S3 **A Small independent validation data set consists of 10 biologically validated target and 10 non-target examples.** This data set is used for finding the optimal parameters of the classifier SVM.
(DOC)

Table S4 **Performance Comparison between MultiMiTar and TargetMiner based on 177 completely independent biologically validated positive test examples.**
(DOC)

Table S5 **Performance Comparison between MultiMiTar and TargetMiner based on 47 completely independent biologically validated negative test examples.**
(DOC)

Table S6 **miRNA-targeting site context specific features (category-wise all the 90 features).**
(DOC)

Table S7 **Performance of MultiMiTar and existing target prediction methods on the 28 experimentally verified Human miRNA-CDKN1A interactions.** In the Table, 1 denotes target and 0 denotes non-target predicted by the respective algorithm.
(DOC)

Table S8 a. Performance of MultiMiTar on KRAS (NM_033360) - hsa-let-7 interactions without considering GU pairing at seed matching site. b. Performance of MultiMiTar on KRAS (NM_033360) - hsa-let-7 interactions with considering single GU pairing at seed matching site.
(DOC)

Dataset S1 **List of downregulated proteins.**
(TXT)

Dataset S2 **List of upregulated proteins.**
(TXT)

Dataset S3 **Predicted miRNA Targets for downregulated data set.**
(TXT)

Dataset S4 **Predicted miRNA Targets for upregulated data set.**
(TXT)
