# Supplementary material for: MultiMiTar: A Novel Multi Objective Optimization based miRNA-Target Prediction Method
Source: PLoS One. 2011 Sep 15;6(9):e24583. doi: 10.1371/journal.pone.0024583 (PMC3174180; doi:10.1371/journal.pone.0024583)
Supplement: Table S1 — List of 289 positive training examples with references. (DOC) [file pone.0024583.s001.doc]

|  | **miRNA** | **Refseq Id** | **References** |
| --- | --- | --- | --- |
| 1 | hsa-let-7a | NM_001039111 | Lin YC et al., 2007 |
| 2 | hsa-let-7a | NM_181833 | Meng et al., 2007 |
| 3 | hsa-let-7a | NM_004985 | Johnson SM et al., 2005 |
| 4 | hsa-let-7a | NM_024674 | Wu L et al., 2006 |
| 5 | hsa-let-7a | NM_003483 | Shell S et al., 2007 |
| 6 | hsa-let-7a | NM_002524 | Johnson SM et al., 2005 |
| 7 | hsa-let-7b | NM_024674 | Kiriakidou et al., 2004 |
| 8 | hsa-let-7b | NM_001259 | Johnson CD et al., 2007 |
| 9 | hsa-let-7b | NM_003483 | Lee YS,Dutta A , 2007 |
| 10 | hsa-let-7b | NM_201567 | Johnson CD et al., 2007 |
| 11 | hsa-let-7c | NM_001039111 | Lin YC et al., 2007 |
| 12 | hsa-let-7c | NM_003483 | Peng Y et al., 2008 |
| 13 | hsa-let-7c | NM_002467 | Koscianska E et al., 2007 |
| 14 | hsa-let-7e | NM_006306 | Kiriakidou et al., 2004 |
| 15 | hsa-let-7e | NM_003483 | Lee YS,Dutta A, 2007 |
| 16 | hsa-let-7g | NM_003483 | Boyerinas et al., 2008 |
| 17 | hsa-let-7g | NM_006546 | Boyerinas B et al., 2008 |
| 18 | hsa-miR-101 | NM_005378 | Lewis BP et al., 2003 |
| 19 | hsa-miR-101 | NM_152998 | Lewis BP et al., 2003 |
| 20 | hsa-miR-103 | NM_015640 | Beitzinger M et al., 2007 |
| 21 | hsa-miR-106a | NM_000321 | Volinia et al., 2006 |
| 22 | hsa-miR-107 | NM_012104 |  |
| 23 | hsa-miR-107 | NM_015640 | Beitzinger M et al., 2007 |
| 24 | hsa-miR-10a | NM_153620 | Garzon et al., 2006 |
| 25 | hsa-miR-10b | NM_002148 | Ma L et al., 2007 |
| 26 | hsa-miR-122 | NM_003045 | Fabani MM et al., 2007 |
| 27 | hsa-miR-125a-5p | NM_024674 | Wu L,Belasco JG, 2005 |
| 28 | hsa-miR-125a-5p | NM_001982 | Scott GK et al., 2007 |
| 29 | hsa-miR-125a-5p | NM_004448 | Scott GK et al., 2007 |
| 30 | hsa-miR-125b | NM_024674 | Wu et al., 2005 |
| 31 | hsa-miR-125b | NM_003901 | Shi XB et al., 2007 |
| 32 | hsa-miR-125b | NM_001806 | Shi XB et al., 2007 |
| 33 | hsa-miR-125b | NM_175709 | Shi XB et al., 2007 |
| 34 | hsa-miR-125b | NM_005324 | Shi XB et al., 2007 |
| 35 | hsa-miR-125b | NM_173473 | Shi XB et al., 2007 |
| 36 | hsa-miR-125b | NM_203339 | Shi XB et al., 2007 |
| 37 | hsa-miR-125b | NM_001982 | Scott GK et al., 2007 |
| 38 | hsa-miR-125b | NM_002644 | Shi XB et al., 2007 |
| 39 | hsa-miR-125b | NM_182511 | Shi XB et al., 2007 |
| 40 | hsa-miR-125b | NM_199332 | Shi XB et al., 2007 |
| 41 | hsa-miR-125b | NM_173843 | Shi XB et al., 2007 |
| 42 | hsa-miR-125b | NM_005105 | Shi XB et al., 2007 |
| 43 | hsa-miR-125b | NM_022121 | Shi XB et al., 2007 |
| 44 | hsa-miR-125b | NM_001188 | Shi XB et al., 2007 |
| 45 | hsa-miR-125b | NM_002167 | Shi XB et al., 2007 |
| 46 | hsa-miR-125b | NM_000499 | Shi XB et al., 2007 |
| 47 | hsa-miR-125b | NM_003538 | Shi XB et al., 2007 |
| 48 | hsa-miR-125b | NM_001007156 | Laneve P et al., 2007 |
| 49 | hsa-miR-125b | NM_014373 | Shi XB et al., 2007 |
| 50 | hsa-miR-125b | NM_004973 | Shi XB et al., 2007 |
| 51 | hsa-miR-125b | NM_001013398 | Shi XB et al., 2007 |
| 52 | hsa-miR-125b | NM_213609 | Shi XB et al., 2007 |
| 53 | hsa-miR-125b | NM_181353 | Shi XB et al., 2007 |
| 54 | hsa-miR-125b | NM_004448 | Scott GK et al., 2007 |
| 55 | hsa-miR-129-5p | NM_177422 | Liao R et al., 2008 |
| 56 | hsa-miR-129-5p | NM_015215 | Liao R et al., 2008 |
| 57 | hsa-miR-130a | NM_019102 | Chen Y et al., 2008 |
| 58 | hsa-miR-130a | NM_005461 | Garzon R et al., 2006 |
| 59 | hsa-miR-130a | NM_005924 | Chen Y et al., 2008 |
| 60 | hsa-miR-130a | NM_000757 | Lewis BP et al., 2003 |
| 61 | hsa-miR-133a | NM_172057 | Xiao J et al., 2007 |
| 62 | hsa-miR-133a | NM_000218 | Luo X et al., 2007 |
| 63 | hsa-miR-133a | NM_001194 | Luo X et al., 2008 |
| 64 | hsa-miR-133b | NM_005029 | Kim J et al., 2007 |
| 65 | hsa-miR-137 | NM_001259 | Kozaki K et al., 2008 |
| 66 | hsa-miR-137 | NM_198256 | Kozaki K et al., 2008 |
| 67 | hsa-miR-137 | NM_006540 | Kozaki K et al., 2008 |
| 68 | hsa-miR-140-5p | NM_003376 | Ye W et al., 2008 |
| 69 | hsa-miR-141 | NM_004898 | Kiriakidou M et al., 2004 |
| 70 | hsa-miR-145 | NM_024615 | Kiriakidou et al., 2004 |
| 71 | hsa-miR-145 | NM_005544 | Shi B et al., 2007 |
| 72 | hsa-miR-147 | NM_003376 | Ye W et al., 2008 |
| 73 | hsa-miR-148a | NM_175850 | Duursma AM et al., 2008 |
| 74 | hsa-miR-148a | NM_175849 | Duursma AM et al., 2008 |
| 75 | hsa-miR-148a | NM_033013 |  |
| 76 | hsa-miR-148b | NM_175850 | Duursma AM et al., 208 |
| 77 | hsa-miR-155 | NM_032049 | Sethupathy P et al., 2007 |
| 78 | hsa-miR-155 | NM_206866 | Skalsky RL et al., 2007 |
| 79 | hsa-miR-15a | NM_000633 | Cimmino A et al., 2005 |
| 80 | hsa-miR-15a | NM_014333 | Calin GA et al., 2008 |
| 81 | hsa-miR-15a | NM_003387 | Calin GA et al., 2008 |
| 82 | hsa-miR-15a | NM_005736 | Calin GA et al., 2008 |
| 83 | hsa-miR-15a | NM_005324 | Calin GA et al., 2008 |
| 84 | hsa-miR-15a | NM_021925 | Calin GA et al., 2008 |
| 85 | hsa-miR-15a | NM_001006605 | Calin GA et al., 2008 |
| 86 | hsa-miR-15a | NM_022173 | Calin GA et al., 2008 |
| 87 | hsa-miR-15a | NM_152729 | Calin GA et al., 2008 |
| 88 | hsa-miR-15a | NM_032303 | Calin GA et al., 2008 |
| 89 | hsa-miR-15a | NM_005316 | Calin GA et al., 2008 |
| 90 | hsa-miR-15a | NM_024759 | Calin GA et al., 2008 |
| 91 | hsa-miR-15a | NM_019083 | Calin GA et al., 2008 |
| 92 | hsa-miR-15a | NM_000365 | Calin GA et al., 2008 |
| 93 | hsa-miR-15a | NM_022353 | Calin GA et al., 2008 |
| 94 | hsa-miR-15a | NM_018307 | Calin GA et al., 2008 |
| 95 | hsa-miR-15a | NM_032124 | Calin GA et al., 2008 |
| 96 | hsa-miR-15a | NM_002228 | Calin GA et al., 2008 |
| 97 | hsa-miR-15a | NM_003930 | Calin GA et al., 2008 |
| 98 | hsa-miR-15a | NM_003376 | Ye W et al., 2008 |
| 99 | hsa-miR-15a | NM_015948 | Calin GA et al., 2008 |
| 100 | hsa-miR-15a | NM_002687 | Calin GA et al., 2008 |
| 101 | hsa-miR-15a | NM_003359 | Calin GA et al., 2008 |
| 102 | hsa-miR-15a | NM_145243 | Calin GA et al., 2008 |
| 103 | hsa-miR-15a | NM_018263 | Calin GA et al., 2008 |
| 104 | hsa-miR-15a | NM_145341 | Calin GA et al., 2008 |
| 105 | hsa-miR-15a | NM_173473 | Calin GA et al., 2008 |
| 106 | hsa-miR-15a | NM_182763 | Calin GA et al., 2008 |
| 107 | hsa-miR-15a | NM_014999 | Calin GA et al., 2008 |
| 108 | hsa-miR-15a | NM_000657 | Calin GA et al., 2008 |
| 109 | hsa-miR-15b | NM_000633 | Xia L et al., 2005 |
| 110 | hsa-miR-16 | NM_003376 | Kiriakidou et al., 2004 |
| 111 | hsa-miR-16 | NM_016140 | Kiriakidou et al., 2004 |
| 112 | hsa-miR-16 | NM_000633 | Xia L et al., 2008 |
| 113 | hsa-miR-16 | NM_003387 | Calin GA et al., 2008 |
| 114 | hsa-miR-16 | NM_014333 | Calin GA et al., 2008 |
| 115 | hsa-miR-16 | NM_005736 | Calin GA et al., 2008 |
| 116 | hsa-miR-16 | NM_005324 | Calin GA et al., 2008 |
| 117 | hsa-miR-16 | NM_032303 | Calin GA et al., 2008 |
| 118 | hsa-miR-16 | NM_021925 | Calin GA et al., 2008 |
| 119 | hsa-miR-16 | NM_001006605 | Calin GA et al., 2008 |
| 120 | hsa-miR-16 | NM_000365 | Calin GA et al., 2008 |
| 121 | hsa-miR-16 | NM_152729 | Calin GA et al., 2008 |
| 122 | hsa-miR-16 | NM_005316 | Calin GA et al., 2008 |
| 123 | hsa-miR-16 | NM_019083 | Calin GA et al., 2008 |
| 124 | hsa-miR-16 | NM_024759 | Calin GA et al., 2008 |
| 125 | hsa-miR-16 | NM_022173 | Calin GA et al., 2008 |
| 126 | hsa-miR-16 | NM_018263 | Calin GA et al., 2008 |
| 127 | hsa-miR-16 | NM_002228 | Calin GA et al., 2008 |
| 128 | hsa-miR-16 | NM_003930 | Calin GA et al., 2008 |
| 129 | hsa-miR-16 | NM_015948 | Calin GA et al., 2008 |
| 130 | hsa-miR-16 | NM_032124 | Calin GA et al., 2008 |
| 131 | hsa-miR-16 | NM_018307 | Calin GA et al., 2008 |
| 132 | hsa-miR-16 | NM_002687 | Calin GA et al., 2008 |
| 133 | hsa-miR-16 | NM_022353 | Calin GA et al., 2008 |
| 134 | hsa-miR-16 | NM_145243 | C,Zanesi N et al., 2008 |
| 135 | hsa-miR-16 | NM_003359 | C,Zanesi N et al., 2008 |
| 136 | hsa-miR-16 | NM_145341 | Calin GA et al., 2008 |
| 137 | hsa-miR-16 | NM_182763 | Calin GA et al., 2008 |
| 138 | hsa-miR-16 | NM_173473 | Calin GA et al., 2008 |
| 139 | hsa-miR-16 | NM_000657 | Calin GA et al., 2008 |
| 140 | hsa-miR-16 | NM_014999 | Calin GA et al., 2008 |
| 141 | hsa-miR-17 | NM_001754 |  |
| 142 | hsa-miR-17 | NM_181659 | Hossain A et al., 2006 |
| 143 | hsa-miR-17 | NM_003376 | Ye W et al., 2008 |
| 144 | hsa-miR-181a | NM_005523 | Naguibneva I et al., 2006 |
| 145 | hsa-miR-181b | NM_003385 | Beveridge NJ et al., 2008 |
| 146 | hsa-miR-181b | NM_021966 | Pekarsky Y et al., 2006 |
| 147 | hsa-miR-182 | NM_015270 | Xu S et al., 2007 |
| 148 | hsa-miR-182 | NM_198159 | Xu S et al., 2007 |
| 149 | hsa-miR-193a-3p | NM_198256 | Kozaki K et al., 2008 |
| 150 | hsa-miR-193a-3p | NM_182763 | Kozaki K et al., 2008 |
| 151 | hsa-miR-193a-3p | NM_005607 | Kozaki K et al., 2008 |
| 152 | hsa-miR-196a | NM_019558 | Yekta S et al., 2004 |
| 153 | hsa-miR-196a | NM_022658 | Yekta S et al., 2004 |
| 154 | hsa-miR-199b-5p | NM_005562 | Kiriakidou M et al., 2004 |
| 155 | hsa-miR-19a | NM_000314 | Lewis et al., 2003 |
| 156 | hsa-miR-200a | NM_014795 | Gregory PA et al., 2008 |
| 157 | hsa-miR-200b | NM_014795 | Gregory PA et al., 2008 |
| 158 | hsa-miR-200b | NM_030751 | Gregory PA et al., 2008 |
| 159 | hsa-miR-200c | NM_030751 | Burk U et al., 2008 |
| 160 | hsa-miR-203 | NM_003722 | Lena AM et al., 2008 |
| 161 | hsa-miR-203 | NM_003955 | Sonkoly E et al., 2007 |
| 162 | hsa-miR-205 | NM_003376 | Ye W et al., 2008 |
| 163 | hsa-miR-205 | NM_014795 | Gregory PA et al., 2008 |
| 164 | hsa-miR-206 | NM_003182 | Greco SJ et al., 2007 |
| 165 | hsa-miR-206 | NM_000165 | Kedde M et al., 2007 |
| 166 | hsa-miR-20a | NM_005225 | O'Donnell KA et al., 2005 |
| 167 | hsa-miR-21 | NM_014456 | Lu Z et al., 2008 |
| 168 | hsa-miR-21 | NM_001018005 | Zhu et al., 2007 |
| 169 | hsa-miR-21 | NM_144949 | Frankel LB et al., 2008 |
| 170 | hsa-miR-21 | NM_005596 | Fujita S et al., 2008 |
| 171 | hsa-miR-21 | NM_181869 | Frankel LB et al., 2008 |
| 172 | hsa-miR-21 | NM_181359 | Frankel LB et al., 2008 |
| 173 | hsa-miR-21 | NM_170709 | Frankel LB et al., 2008 |
| 174 | hsa-miR-21 | NM_018593 | Frankel LB et al., 2008 |
| 175 | hsa-miR-21 | NM_001259 | Frankel LB et al., 2007 |
| 176 | hsa-miR-21 | NM_001204 | Frankel LB et al., 2007 |
| 177 | hsa-miR-21 | NM_002639 | Zhu S et al., 2008 |
| 178 | hsa-miR-21 | NM_005734 | Frankel LB et al., 2008 |
| 179 | hsa-miR-21 | NM_014454 | Frankel LB et al., 2007 |
| 180 | hsa-miR-210 | NM_004952 | Fasanaro P et al., 2008 |
| 181 | hsa-miR-212 | NM_175610 | Tang Y et al., 2008 |
| 182 | hsa-miR-214 | NM_000314 | Yang H et al., 2008 |
| 183 | hsa-miR-218 | NM_001017402 | Martinez I et al., 2008 |
| 184 | hsa-miR-22 | NM_000125 | McManus MT, 2008 |
| 185 | hsa-miR-22 | NM_001001929 |  |
| 186 | hsa-miR-221 | NM_000222 | Felli et al., 2005 |
| 187 | hsa-miR-221 | NM_004064 | Kedde M et al., 2007/ Felicetti F et al., 2008 |
| 188 | hsa-miR-222 | NM_000222 | Felli et al., 2005 |
| 189 | hsa-miR-222 | NM_004064 | Galardi et al, 2007 |
| 190 | hsa-miR-223 | NM_005595 | Fazi F et al., 2005 |
| 191 | hsa-miR-23a | NM_024909 | Kiriakidou et al., 2004 |
| 192 | hsa-miR-23a | NM_004575 | Lewis BP et al., 2003 |
| 193 | hsa-miR-23a | NM_005524 | Kawasaki H et al., 2004 |
| 194 | hsa-miR-24 | NM_000791 | Mishra et al., 2007 |
| 195 | hsa-miR-24 | NM_004302 | Wang Q et al., 2008 |
| 196 | hsa-miR-26a | NM_005900 | Luzi E et al., 2008 |
| 197 | hsa-miR-26a | NM_002655 | Volinia et al., 2006 |
| 198 | hsa-miR-26a | NM_003242 | Volinia S et al., 2006 |
| 199 | hsa-miR-27b | NM_000104 | Tsuchiya et al., 2006 |
| 200 | hsa-miR-29a | NM_175848 | Fabbri M et al., 2007 |
| 201 | hsa-miR-29a | NM_022552 | Fabbri M et al., 2007 |
| 202 | hsa-miR-29a | NM_003118 | Sengupta S et al., 2008 |
| 203 | hsa-miR-29b | NM_175848 | Fabbri M et al., 2007 |
| 204 | hsa-miR-29b | NM_022552 | Fabbri M et al., 2007 |
| 205 | hsa-miR-29b | NM_021960 | Mott jl et al., 2007 |
| 206 | hsa-miR-29b | NM_021966 | Pekarsky Y et al., 2006 |
| 207 | hsa-miR-29c | NM_175848 | Fabbri M et al., 2007 |
| 208 | hsa-miR-29c | NM_022552 | Fabbri M et al., 2007 |
| 209 | hsa-miR-29c | NM_003211 | Sengupta S et al., 2008 |
| 210 | hsa-miR-29c | NM_001855 | Sengupta S et al., 2008 |
| 211 | hsa-miR-29c | NM_000090 | Sengupta S et al., 2008 |
| 212 | hsa-miR-29c | NM_002293 | Wang X and Wang X 2006 |
| 213 | hsa-miR-29c | NM_001845 | Sengupta S et al., 2008 |
| 214 | hsa-miR-29c | NM_000089 | Sengupta S et al., 2008 |
| 215 | hsa-miR-29c | NM_000138 | Sengupta S et al., 2008 |
| 216 | hsa-miR-29c | NM_001846 | Sengupta S et al., 2008 |
| 217 | hsa-miR-29c | NM_000088 | Sengupta S et al., 2008 |
| 218 | hsa-miR-30a* | NM_003983 | Nakamoto M et al., 2005 |
| 219 | hsa-miR-30a* | NM_025222 | Nakamoto M et al., 2005 |
| 220 | hsa-miR-30a* | NM_003246 | Nakamoto M et al., 2005 |
| 221 | hsa-miR-30a* | NM_017599 | Nakamoto M, et al., 2005 |
| 222 | hsa-miR-30a* | NM_001259 | Nakamoto M et al., 2005 |
| 223 | hsa-miR-30a* | NM_006009 | Nakamoto M et al., 2005 |
| 224 | hsa-miR-34a | NM_001949 | Welch et al., 2007 |
| 225 | hsa-miR-34a | NM_000633 | Bommer GT et al., 2007 |
| 226 | hsa-miR-34a | NM_003376 | Ye W et al., 2008 |
| 227 | hsa-miR-34a | NM_005618 | Lewis BP et al., 2003 |
| 228 | hsa-miR-34a | NM_017617 | Lewis BP et al., 2003 |
| 229 | hsa-miR-34a | NM_005378 | Wei JS et al., 2008 |
| 230 | hsa-miR-34b* | NM_003376 | Ye W et al., 2008 |
| 231 | hsa-miR-370 | NM_005204 | Meng F et al., 2008 |
| 232 | hsa-miR-372 | NM_014572 | Voorhoeve et al., 2006 |
| 233 | hsa-miR-372 | NM_003376 | Ye W et al., 2008 |
| 234 | hsa-miR-373 | NM_015455 | Lim et al., 2005 |
| 235 | hsa-miR-373 | NM_152835 | Lim et al., 2005 |
| 236 | hsa-miR-373 | NM_015076 | Lim et al., 2005 |
| 237 | hsa-miR-373 | NM_016603 | Lim et al., 2005 |
| 238 | hsa-miR-373 | NM_021914 | Lim et al., 2005 |
| 239 | hsa-miR-373 | NM_004896 | Lim et al., 2005 |
| 240 | hsa-miR-373 | NM_153042 | Lim et al., 2005 |
| 241 | hsa-miR-373 | NM_152261 | Lim et al., 2005 |
| 242 | hsa-miR-373 | NM_016343 | Lim et al., 2005 |
| 243 | hsa-miR-373 | NM_006282 | Lim et al., 2005 |
| 244 | hsa-miR-373 | NM_004427 | Lim et al., 2005 |
| 245 | hsa-miR-373 | NM_152345 | Lim et al., 2005 |
| 246 | hsa-miR-373 | NM_153369 | Lim et al., 2005 |
| 247 | hsa-miR-373 | NM_013446 | Lim et al., 2005 |
| 248 | hsa-miR-373 | NM_178818 | Lim et al., 2005 |
| 249 | hsa-miR-373 | NM_016019 | Lim et al., 2005 |
| 250 | hsa-miR-373 | NM_004233 | Lim et al., 2005 |
| 251 | hsa-miR-373 | NM_003764 | Lim et al., 2005 |
| 252 | hsa-miR-373 | NM_003981 | Lim et al., 2005 |
| 253 | hsa-miR-373 | NM_004404 | Lim et al., 2005 |
| 254 | hsa-miR-373 | NM_003223 | Lim et al., 2005 |
| 255 | hsa-miR-373 | NM_014051 | Lim et al., 2005 |
| 256 | hsa-miR-373 | NM_144596 | Lim et al., 2005 |
| 257 | hsa-miR-373 | NM_019555 | Lim et al., 2005 |
| 258 | hsa-miR-373 | NM_145048 | Lim et al., 2005 |
| 259 | hsa-miR-373 | NM_144563 | Lim et al., 2005 |
| 260 | hsa-miR-373 | NM_013448 | Lim et al., 2005 |
| 261 | hsa-miR-373 | NM_016133 | Lim et al., 2005 |
| 262 | hsa-miR-373 | NM_016433 | Lim et al., 2005 |
| 263 | hsa-miR-373 | NM_003816 | Lim et al., 2005 |
| 264 | hsa-miR-373 | NM_005573 | Lim et al., 2005 |
| 265 | hsa-miR-373 | NM_015853 | Lim et al., 2005 |
| 266 | hsa-miR-373 | NM_018421 | Lim et al., 2005 |
| 267 | hsa-miR-373 | NM_173647 | Lim et al., 2005 |
| 268 | hsa-miR-373 | NM_025205 | Lim et al., 2005 |
| 269 | hsa-miR-373 | NM_021633 | Lim et al., 2005 |
| 270 | hsa-miR-373 | NM_018492 | Lim et al., 2005 |
| 271 | hsa-miR-373 | NM_014572 | Voorhoeve et al., 2006 |
| 272 | hsa-miR-373 | NM_001001392 | Huang Q et al., 2008 |
| 273 | hsa-miR-376a* | NM_004768 | Kawahara Y et al., 2007 |
| 274 | hsa-miR-376a* | NM_003051 | Kawahara Y et al., 2007 |
| 275 | hsa-miR-376a* | NM_003318 | Kawahara Y et al., 2007 |
| 276 | hsa-miR-378* | NM_016169 | Lee DY et al., 2007 |
| 277 | hsa-miR-424 | NM_005595 | Rosa A et al., 2007 |
| 278 | hsa-miR-433 | NM_019851 | Wang G et al., 2008 |
| 279 | hsa-miR-504 | NM_003376 | Ye W et al., 2008 |
| 280 | hsa-miR-520g | NM_003376 | Ye W et al., 2008 |
| 281 | hsa-miR-520h | NM_004827 | Liao R et al., 2008 |
| 282 | hsa-miR-520h | NM_003376 | Ye W et al., 2008 |
| 283 | hsa-miR-7 | NM_003749 | Kefas B et al., 2008 |
| 284 | hsa-miR-7 | NM_005228 | Kefas B et al., 2008 |
| 285 | hsa-miR-7 | NM_005544 | Kefas B et al., 2008 |
| 286 | hsa-miR-9 | NM_001007156 | Laneve P et al., 2007 |
| 287 | hsa-miR-96 | NM_015270 | Xu S et al., 2007 |
| 288 | hsa-miR-96 | NM_198159 | Xu S et al., 2007 |
| 289 | hsa-miR-98 | NM_003483 | Hebert et al., 2007 |
