# Supplementary material for: MultiMiTar: A Novel Multi Objective Optimization based miRNA-Target Prediction Method
Source: PLoS One. 2011 Sep 15;6(9):e24583. doi: 10.1371/journal.pone.0024583 (PMC3174180; doi:10.1371/journal.pone.0024583)
Supplement: Table S2 — List of 289 tissue specific negative training examples. (DOC) [file pone.0024583.s002.doc]

|  | **miRNA** | **Refseq ID** | **Tissue Type** |  | **miRNA** | **Refseq ID** | **Tissue Type** |
| --- | --- | --- | --- | --- | --- | --- | --- |
| 1 | hsa-let-7g | NM_005546 | Thymus | 146 | hsa-miR-204 | NM_178424 | Testis |
| 2 | hsa-let-7g | NM_014716 | Thymus | 147 | hsa-miR-204 | NM_014384 | Kidney |
| 3 | hsa-let-7i | NM_001152 | Thymus | 148 | hsa-miR-204 | NM_000159 | Kidney |
| 4 | hsa-let-7i | NM_014716 | Thymus | 149 | hsa-miR-204 | NM_013976 | Kidney |
| 5 | hsa-let-7i | NM_004456 | Thymus | 150 | hsa-miR-204 | NM_002361 | Kidney |
| 6 | hsa-let-7i | NM_152998 | Thymus | 151 | hsa-miR-204 | NM_000294 | Testis |
| 7 | hsa-miR-1 | NM_002812 | Heart | 152 | hsa-miR-205 | NM_005978 | Prostate |
| 8 | hsa-miR-1 | NM_002078 | Prostate | 153 | hsa-miR-205 | NM_003890 | Prostate, Thymus |
| 9 | hsa-miR-1 | NM_000461 | Heart | 154 | hsa-miR-205 | NM_001886 | Prostate |
| 10 | hsa-miR-1 | NM_000481 | Heart | 155 | hsa-miR-205 | NM_030960 | Prostate |
| 11 | hsa-miR-1 | NM_003613 | Heart | 156 | hsa-miR-205 | NM_004103 | Thymus |
| 12 | hsa-miR-103 | NM_003165 | Brain | 157 | hsa-miR-205 | NM_173174 | Thymus |
| 13 | hsa-miR-103 | NM_016453 | Brain | 158 | hsa-miR-205 | NM_173176 | Thymus |
| 14 | hsa-miR-103 | NM_184231 | Brain | 159 | hsa-miR-205 | NM_030770 | Prostate |
| 15 | hsa-miR-103 | NM_000067 | Brain | 160 | hsa-miR-205 | NM_030775 | Prostate |
| 16 | hsa-miR-103 | NM_014575 | Brain | 161 | hsa-miR-22 | NM_005855 | Liver |
| 17 | hsa-miR-103 | NM_001094 | Brain | 162 | hsa-miR-22 | NM_022449 | Liver |
| 18 | hsa-miR-124 | NM_019555 | Brain | 163 | hsa-miR-22 | NM_001013436 | Liver |
| 19 | hsa-miR-124 | NM_006527 | Brain | 164 | hsa-miR-22 | NM_001013440 | Liver |
| 20 | hsa-miR-124 | NM_015320 | Brain | 165 | hsa-miR-22 | NM_001003954 | Liver |
| 21 | hsa-miR-124 | NM_002436 | Brain | 166 | hsa-miR-22 | NM_004306 | Liver |
| 22 | hsa-miR-124 | NM_003458 | Brain | 167 | hsa-miR-22 | NM_005700 | Liver |
| 23 | hsa-miR-132 | NM_016310 | Brain | 168 | hsa-miR-22 | NM_005029 | Liver |
| 24 | hsa-miR-132 | NM_000320 | Brain | 169 | hsa-miR-22 | NM_000017 | Liver |
| 25 | hsa-miR-132 | NM_001819 | Brain | 170 | hsa-miR-22 | NM_001144 | Liver |
| 26 | hsa-miR-132 | NM_004505 | Brain | 171 | hsa-miR-22 | NM_005258 | Liver |
| 27 | hsa-miR-132 | NM_182470 | Brain | 172 | hsa-miR-221 | NM_024663 | Brain |
| 28 | hsa-miR-137 | NM_005574 | Brain, Lung | 173 | hsa-miR-221 | NM_014303 | Brain |
| 29 | hsa-miR-137 | NM_005539 | Brain | 174 | hsa-miR-221 | NM_000251 | Brain |
| 30 | hsa-miR-137 | NM_002436 | Brain | 175 | hsa-miR-222 | NM_000251 | Brain |
| 31 | hsa-miR-137 | NM_003763 | Brain | 176 | hsa-miR-222 | NM_004541 | Brain |
| 32 | hsa-miR-137 | NM_001001433 | Brain | 177 | hsa-miR-222 | NM_014820 | Brain |
| 33 | hsa-miR-137 | NM_024663 | Brain | 178 | hsa-miR-23a | NM_003890 | Lung, Thymus |
| 34 | hsa-miR-138 | NM_014110 | Brain | 179 | hsa-miR-23a | NM_005720 | Lung |
| 35 | hsa-miR-138 | NM_003592 | Brain | 180 | hsa-miR-23a | NM_003255 | Lung |
| 36 | hsa-miR-138 | NM_003325 | Brain | 181 | hsa-miR-23a | NM_018961 | Thymus |
| 37 | hsa-miR-138 | NM_002109 | Brain | 182 | hsa-miR-23b | NM_000191 | Heart |
| 38 | hsa-miR-138 | NM_002738 | Brain | 183 | hsa-miR-23b | NM_002488 | Heart |
| 39 | hsa-miR-138 | NM_002695 | Brain | 184 | hsa-miR-23b | NM_000435 | Heart |
| 40 | hsa-miR-138 | NM_004564 | Brain | 185 | hsa-miR-23b | NM_001163 | Heart |
| 41 | hsa-miR-143 | NM_001185 | Prostate | 186 | hsa-miR-23b | NM_017533 | Heart |
| 42 | hsa-miR-143 | NM_004126 | Uterus | 187 | hsa-miR-23b | NM_005001 | Heart |
| 43 | hsa-miR-143 | NM_021910 | Prostate | 188 | hsa-miR-24 | NM_004750 | Placenta |
| 44 | hsa-miR-143 | NM_000867 | Prostate, Uterus | 189 | hsa-miR-24 | NM_002019 | Placenta |
| 45 | hsa-miR-145 | NM_005978 | Prostate | 190 | hsa-miR-24 | NM_017459 | Placenta |
| 46 | hsa-miR-145 | NM_015392 | Prostate, Uterus | 191 | hsa-miR-24 | NM_002403 | Placenta |
| 47 | hsa-miR-145 | NM_000299 | Prostate | 192 | hsa-miR-24 | NM_002084 | Placenta |
| 48 | hsa-miR-145 | NM_003008 | Prostate | 193 | hsa-miR-24 | NM_001124 | Placenta |
| 49 | hsa-miR-145 | NM_005795 | Uterus | 194 | hsa-miR-24 | NM_004995 | Placenta |
| 50 | hsa-miR-145 | NM_004755 | Uterus | 195 | hsa-miR-24 | NM_002964 | Placenta |
| 51 | hsa-miR-145 | NM_004672 | Prostate, Uterus | 196 | hsa-miR-24 | NM_004429 | Placenta |
| 52 | hsa-miR-145 | NM_000350 | Prostate | 197 | hsa-miR-26a | NM_000719 | Uterus |
| 53 | hsa-miR-145 | NM_004532 | Prostate | 198 | hsa-miR-26a | NM_005257 | Uterus |
| 54 | hsa-miR-145 | NM_004785 | Prostate | 199 | hsa-miR-26a | NM_018222 | Uterus |
| 55 | hsa-miR-145 | NM_021910 | Prostate | 200 | hsa-miR-26a | NM_004684 | Uterus |
| 56 | hsa-miR-145 | NM_001039550 | Prostate | 201 | hsa-miR-26b | NM_004684 | Uterus |
| 57 | hsa-miR-153 | NM_005165 | Brain | 202 | hsa-miR-26b | NM_017426 | Uterus |
| 58 | hsa-miR-153 | NM_001092 | Brain | 203 | hsa-miR-26b | NM_018222 | Uterus |
| 59 | hsa-miR-153 | NM_015185 | Brain | 204 | hsa-miR-26b | NM_000210 | Uterus |
| 60 | hsa-miR-153 | NM_004644 | Brain | 205 | hsa-miR-27a | NM_005545 | Uterus |
| 61 | hsa-miR-153 | NM_005648 | Brain | 206 | hsa-miR-27a | NM_201526 | Uterus |
| 62 | hsa-miR-153 | NM_006158 | Brain | 207 | hsa-miR-27a | NM_001001392 | Uterus |
| 63 | hsa-miR-153 | NM_001201 | Brain, Pancreas | 208 | hsa-miR-27a | NM_001001390 | Uterus |
| 64 | hsa-miR-153 | NM_000320 | Brain | 209 | hsa-miR-27a | NM_001001391 | Uterus |
| 65 | hsa-miR-153 | NM_005271 | Brain | 210 | hsa-miR-27a | NM_001001389 | Uterus |
| 66 | hsa-miR-153 | NM_005010 | Brain | 211 | hsa-miR-27a | NM_000610 | Uterus |
| 67 | hsa-miR-153 | NM_001037132 | Brain | 212 | hsa-miR-27a | NM_198177 | Uterus |
| 68 | hsa-miR-153 | NM_001037133 | Brain | 213 | hsa-miR-27a | NM_198159 | Uterus |
| 69 | hsa-miR-153 | NM_176795 | Brain | 214 | hsa-miR-27a | NM_198158 | Uterus |
| 70 | hsa-miR-153 | NM_005343 | Brain | 215 | hsa-miR-27a | NM_000248 | Uterus |
| 71 | hsa-miR-153 | NM_006176 | Brain | 216 | hsa-miR-27a | NM_198178 | Uterus |
| 72 | hsa-miR-153 | NM_016237 | Brain | 217 | hsa-miR-27a | NM_198256 | Uterus |
| 73 | hsa-miR-153 | NM_003367 | Brain | 218 | hsa-miR-29a | NM_001849 | Uterus |
| 74 | hsa-miR-153 | NM_207291 | Brain | 219 | hsa-miR-29a | NM_005157 | Uterus |
| 75 | hsa-miR-153 | NM_004128 | Pancreas | 220 | hsa-miR-29a | NM_001856 | Uterus |
| 76 | hsa-miR-155 | NM_000850 | Kidney | 221 | hsa-miR-29a | NM_002403 | Uterus |
| 77 | hsa-miR-155 | NM_001122 | Kidney | 222 | hsa-miR-29a | NM_017459 | Uterus |
| 78 | hsa-miR-155 | NM_152869 | Kidney | 223 | hsa-miR-29a | NM_021738 | Uterus |
| 79 | hsa-miR-155 | NM_004683 | Kidney | 224 | hsa-miR-29a | NM_003174 | Uterus |
| 80 | hsa-miR-15a | NM_001003941 | Heart | 225 | hsa-miR-29a | NM_014296 | Uterus |
| 81 | hsa-miR-15a | NM_002541 | Heart | 226 | hsa-miR-29a | NM_014211 | Uterus |
| 82 | hsa-miR-15a | NM_006254 | Bone marrow | 227 | hsa-miR-29a | NM_016831 | Uterus |
| 83 | hsa-miR-15a | NM_212539 | Bone marrow | 228 | hsa-miR-29b | NM_201539 | Brain |
| 84 | hsa-miR-15a | NM_000803 | Heart | 229 | hsa-miR-29b | NM_201540 | Brain |
| 85 | hsa-miR-15a | NM_000552 | Heart | 230 | hsa-miR-29b | NM_201535 | Brain |
| 86 | hsa-miR-15a | NM_006412 | Heart | 231 | hsa-miR-29b | NM_201537 | Brain |
| 87 | hsa-miR-15a | NM_000286 | Bone marrow | 232 | hsa-miR-29b | NM_182764 | Brain |
| 88 | hsa-miR-15a | NM_001024382 | Bone marrow | 233 | hsa-miR-29b | NM_002811 | Brain |
| 89 | hsa-miR-15a | NM_000190 | Bone marrow | 234 | hsa-miR-29b | NM_000067 | Brain |
| 90 | hsa-miR-15a | NM_001040031 | Bone marrow, Heart | 235 | hsa-miR-29b | NM_005175 | Brain |
| 91 | hsa-miR-15a | NM_001002027 | Heart | 236 | hsa-miR-29b | NM_001002027 | Brain |
| 92 | hsa-miR-15a | NM_005175 | Heart | 237 | hsa-miR-29b | NM_002079 | Brain |
| 93 | hsa-miR-15a | NM_002134 | Heart | 238 | hsa-miR-30c | NM_003891 | Heart. Kidney |
| 94 | hsa-miR-15a | NM_005675 | Heart | 239 | hsa-miR-30c | NM_002084 | Heart. Kidney |
| 95 | hsa-miR-15a | NM_002250 | Bone marrow, Heart | 240 | hsa-miR-30c | NM_000124 | Heart |
| 96 | hsa-miR-15a | NM_018696 | Heart | 241 | hsa-miR-30c | NM_003569 | Kidney |
| 97 | hsa-miR-15a | NM_005529 | Heart | 242 | hsa-miR-30c | NM_005956 | Kidney |
| 98 | hsa-miR-190 | NM_013445 | Brain | 243 | hsa-miR-30c | NM_000192 | Heart |
| 99 | hsa-miR-190 | NM_003350 | Brain | 244 | hsa-miR-30c | NM_181486 | Heart |
| 100 | hsa-miR-190 | NM_003430 | Brain | 245 | hsa-miR-30c | NM_005550 | Kidney |
| 101 | hsa-miR-190 | NM_006409 | Brain | 246 | hsa-miR-30c | NM_002633 | Heart |
| 102 | hsa-miR-191 | NM_003846 | Brain | 247 | hsa-miR-30c | NM_000727 | Heart |
| 103 | hsa-miR-191 | NM_001014444 | Brain | 248 | hsa-miR-30c | NM_000696 | Kidney |
| 104 | hsa-miR-191 | NM_001888 | Brain | 249 | hsa-miR-30c | NM_002631 | Kidney |
| 105 | hsa-miR-191 | NM_002126 | Brain | 250 | hsa-miR-30c | NM_002560 | Kidney |
| 106 | hsa-miR-191 | NM_002109 | Brain | 251 | hsa-miR-345 | NM_018962 | Bone marrow |
| 107 | hsa-miR-191 | NM_022003 | Brain | 252 | hsa-miR-7 | NM_006343 | Brain, Adrenal |
| 108 | hsa-miR-191 | NM_004420 | Brain | 253 | hsa-miR-7 | NM_139343 | Brain, Adrenal |
| 109 | hsa-miR-196b | NM_014164 | Bone marrow | 254 | hsa-miR-7 | NM_139348 | Brain, Adrenal |
| 110 | hsa-miR-196b | NM_144779 | Bone marrow | 255 | hsa-miR-7 | NM_139346 | Brain, Adrenal |
| 111 | hsa-miR-196b | NM_001870 | Bone marrow | 256 | hsa-miR-7 | NM_002695 | Brain |
| 112 | hsa-miR-196b | NM_003120 | Bone marrow | 257 | hsa-miR-7 | NM_021727 | Brain, Adrenal |
| 113 | hsa-miR-200a | NM_004988 | Pancreas, Kidney | 258 | hsa-miR-7 | NM_002390 | Brain, Adrenal |
| 114 | hsa-miR-200a | NM_002674 | Kidney | 259 | hsa-miR-7 | NM_005872 | Brain |
| 115 | hsa-miR-200a | NM_021804 | Kidney | 260 | hsa-miR-7 | NM_000835 | Brain |
| 116 | hsa-miR-200a | NM_030765 | Kidney | 261 | hsa-miR-7 | NM_002436 | Brain |
| 117 | hsa-miR-200a | NM_002020 | Kidney | 262 | hsa-miR-7 | NM_022169 | Brain, Adrenal |
| 118 | hsa-miR-200a | NM_182925 | Kidney | 263 | hsa-miR-7 | NM_000218 | Adrenal |
| 119 | hsa-miR-200a | NM_014384 | Kidney | 264 | hsa-miR-7 | NM_004322 | Brain |
| 120 | hsa-miR-200a | NM_004170 | Kidney | 265 | hsa-miR-7 | NM_004378 | Brain |
| 121 | hsa-miR-203 | NM_002087 | Lung | 266 | hsa-miR-9 | NM_003458 | Brain |
| 122 | hsa-miR-204 | NM_006066 | Kidney | 267 | hsa-miR-9 | NM_003165 | Brain |
| 123 | hsa-miR-204 | NM_004505 | Testis | 268 | hsa-miR-9 | NM_001032221 | Brain |
| 124 | hsa-miR-204 | NM_153285 | Kidney | 269 | hsa-miR-9 | NM_005736 | Brain |
| 125 | hsa-miR-204 | NM_153283 | Kidney | 270 | hsa-miR-9 | NM_004569 | Brain |
| 126 | hsa-miR-204 | NM_153281 | Kidney | 271 | hsa-miR-9 | NM_199295 | Brain |
| 127 | hsa-miR-204 | NM_014236 | Kidney, Testis | 272 | hsa-miR-9 | NM_004505 | Brain |
| 128 | hsa-miR-204 | NM_003041 | Kidney, Testis | 273 | hsa-miR-9 | NM_000255 | Brain |
| 129 | hsa-miR-204 | NM_182710 | Testis | 274 | hsa-miR-9 | NM_153500 | Brain |
| 130 | hsa-miR-204 | NM_015971 | Kidney, Testis | 275 | hsa-miR-9 | NM_006549 | Brain |
| 131 | hsa-miR-204 | NM_000035 | Kidney | 276 | hsa-miR-9 | NM_172226 | Brain |
| 132 | hsa-miR-204 | NM_004790 | Kidney | 277 | hsa-miR-9 | NM_153499 | Brain |
| 133 | hsa-miR-204 | NM_005605 | Testis | 278 | hsa-miR-9 | NM_172216 | Brain |
| 134 | hsa-miR-204 | NM_153766 | Kidney | 279 | hsa-miR-9 | NM_172214 | Brain |
| 135 | hsa-miR-204 | NM_000220 | Kidney | 280 | hsa-miR-9 | NM_172215 | Brain |
| 136 | hsa-miR-204 | NM_153765 | Kidney | 281 | hsa-miR-9 | NM_020184 | Brain |
| 137 | hsa-miR-204 | NM_153767 | Kidney | 282 | hsa-miR-9 | NM_014292 | Brain |
| 138 | hsa-miR-204 | NM_153764 | Kidney | 283 | hsa-miR-9 | NM_002300 | Brain |
| 139 | hsa-miR-204 | NM_004110 | Testis | 284 | hsa-miR-9 | NM_005891 | brain |
| 140 | hsa-miR-204 | NM_000520 | Kidney | 285 | hsa-mir-99a | NM_004341 | Uterus |
| 141 | hsa-miR-204 | NM_002428 | Kidney, Testis | 286 | hsa-miR-99b | NM_000432 | Heart |
| 142 | hsa-miR-204 | NM_002147 | Kidney | 287 | hsa-miR-99b | NM_005498 | Heart |
| 143 | hsa-miR-204 | NM_002153 | Kidney | 288 | hsa-miR-99b | NM_014228 | Heart |
| 144 | hsa-miR-204 | NM_002980 | Kidney, Testis | 289 | hsa-miR-99b | NM_002165 | Heart |
| 145 | hsa-miR-204 | NM_005074 | Kidney |  |  |  |  |
