# Supplementary material for: MultiMiTar: A Novel Multi Objective Optimization based miRNA-Target Prediction Method
Source: PLoS One. 2011 Sep 15;6(9):e24583. doi: 10.1371/journal.pone.0024583 (PMC3174180; doi:10.1371/journal.pone.0024583)
Supplement: Table S3 — A Small independent validation data set consists of 10 biologically validated target and 10 non-target examples. This data set is used for finding the optimal parameters of the classifier SVM. (DOC) [file pone.0024583.s003.doc]

|  | **miRNA** | **mRNA** | **Biologically validated** |
| --- | --- | --- | --- |
| 1 | hsa-miR-140-3p | NM_006037 | Target |
| 2 | hsa-miR-1 | NM_000165 | Target |
| 3 | hsa-miR-1 | NM_003769 | Target |
| 4 | hsa-miR-1 | NM_006148 | Target |
| 5 | hsa-miR-1 | NM_003417 | Target |
| 6 | hsa-miR-1 | NM_006454 | Target |
| 7 | hsa-miR-1 | NM_007081 | Target |
| 8 | hsa-miR-1 | NM_003290 | Target |
| 9 | hsa-miR-1 | NM_019114 | Target |
| 10 | hsa-miR-124 | NM_000696 | Target |
| 11 | hsa-let-7b | NM_144563 | Non-Target |
| 12 | hsa-miR-29 | NM_000883 | Non-Target |
| 13 | hsa-miR-24 | NM_014730 | Non-Target |
| 14 | hsa-let-7e | NM_003758 | Non-Target |
| 15 | hsa-miR-19a | NM_001110792 | Non-Target |
| 16 | mmu-miR-145 | NM_016963 | Non-Target |
| 17 | mmu-miR-375 | NM_013464 | Non-Target |
| 18 | mmu-miR-103 | NM_010271 | Non-Target |
| 19 | mmu-miR-1 | NM_184052 | Non-Target |
| 20 | mmu-miR-215 | NM_025656 | Non-Target |
