# Supplementary material for: MultiMiTar: A Novel Multi Objective Optimization based miRNA-Target Prediction Method
Source: PLoS One. 2011 Sep 15;6(9):e24583. doi: 10.1371/journal.pone.0024583 (PMC3174180; doi:10.1371/journal.pone.0024583)
Supplement: Table S4 — Performance Comparison between MultiMiTar and TargetMiner based on 177 completely independent biologically validated positive test examples. (DOC) [file pone.0024583.s004.doc]

|  | **miRNA** | **mRNA** | **References** | **TargetMiner Prediction** | **MultiMiTar**  **Prediction score** |
| --- | --- | --- | --- | --- | --- |
| 1 | hsa-miR-1 | NM_020347 | Lim et al., 2005 | Target | 0.271926 |
| 2 | hsa-miR-1 | NM_005715 | Lim et al., 2005 | Target | 0.358859 |
| 3 | hsa-miR-1 | NM_145257 | Lim et al., 2005 | Target | 0.359729 |
| 4 | hsa-miR-1 | NM_002822 | Lim et al., 2005 | Target | 0.270383 |
| 5 | hsa-miR-1 | NM_004520 | Lim et al., 2005 | Target | 0.138471 |
| 6 | hsa-miR-1 | NM_018362 | Lim et al., 2005 | Target | 0.37639 |
| 7 | hsa-miR-1 | NM_016271 | Lim et al., 2005 | Target | 0.172932 |
| 8 | hsa-miR-1 | NM_000362 | Lim et al., 2005 | Target | 0.307785 |
| 9 | hsa-miR-1 | NM_001111 | Lim et al., 2005 | Target | 0.184201 |
| 10 | hsa-miR-1 | NM_014445 | Lim et al., 2005 | Target | 0.359814 |
| 11 | hsa-miR-1 | NM_000891 | Lim et al., 2005 | Target | 0.288883 |
| 12 | hsa-miR-1 | NM_017542 | Lim et al., 2005 | Target | 0.226452 |
| 13 | hsa-miR-1 | NM_005324 | Lim et al., 2005 | Target | 0.233261 |
| 14 | hsa-miR-1 | NM_015271 | Lim et al., 2005 | Target | 0.358952 |
| 15 | hsa-miR-1 | NM_019004 | Lim et al., 2005 | Target | 0.220038 |
| 16 | hsa-miR-1 | NM_019063 | Lim et al., 2005 | Target | 0.40507 |
| 17 | hsa-miR-1 | NM_015455 | Lim et al., 2005 | Target | 0.332442 |
| 18 | hsa-miR-1 | NM_014904 | Lim et al., 2005 | Target | 0.206111 |
| 19 | hsa-miR-1 | NM_000161 | Lim et al., 2005 | Target | 0.337989 |
| 20 | hsa-miR-1 | NM_003203 | Lim et al., 2005 | Target | 0.16256 |
| 21 | hsa-miR-1 | NM_019027 | Lim et al., 2005 | Target | 0.380357 |
| 22 | hsa-miR-1 | NM_152282 | Lim et al., 2005 | Target | 0.184633 |
| 23 | hsa-miR-1 | NM_024738 | Lim et al., 2005 | Target | 0.322085 |
| 24 | hsa-miR-1 | NM_145341 | Lim et al., 2005 | Target | 0.330396 |
| 25 | hsa-miR-1 | NM_175866 | Lim et al., 2005 | Target | 0.204097 |
| 26 | hsa-miR-1 | NM_000219 | Lim et al., 2005 | Target | 0.265204 |
| 27 | hsa-miR-1 | NM_170735 | Lim et al., 2005 | Target | 0.349518 |
| 28 | hsa-miR-1 | NM_014918 | Lim et al., 2005 | Target | 0.286438 |
| 29 | hsa-miR-1 | NM_018448 | Lim et al., 2005 | Target | 0.15513 |
| 30 | hsa-miR-1 | NM_004815 | Lim et al., 2005 | Target | 0.38964 |
| 31 | hsa-miR-1 | NM_000274 | Lim et al., 2005 | Target | 0.096386 |
| 32 | hsa-miR-1 | NM_014937 | Lim et al., 2005 | Target | 0.205724 |
| 33 | hsa-miR-1 | NM_014408 | Lim et al., 2005 | Target | 0.21489 |
| 34 | hsa-miR-1 | NM_013412 | Lim et al., 2005 | Non-Target | 0.021854 |
| 35 | hsa-miR-1 | NM_005034 | Lim et al., 2005 | Non-Target | 0.005071 |
| 36 | hsa-miR-1 | NM_002639 | Lim et al., 2005 | Non-Target | Non-Target |
| 37 | hsa-miR-1 | NM_018319 | Lim et al., 2005 | Non-Target | Non-Target |
| 38 | hsa-miR-1 | NM_000270 | Lim et al., 2005 | Non-Target | Non-Target |
| 39 | hsa-miR-1 | NM_018413 | Lim et al., 2005 | Non-Target | Non-Target |
| 40 | hsa-miR-1 | NM_006367 | Lim et al., 2005 | Non-Target | Non-Target |
| 41 | hsa-miR-1 | NM_003564 | Lim et al., 2005 | Non-Target | 0.043712 |
| 42 | hsa-miR-1 | NM_022075 | Lim et al., 2005 | Non-Target | 0.094313 |
| 43 | hsa-miR-1 | NM_001829 | Lim et al., 2005 | Non-Target | Non-Target |
| 44 | hsa-miR-1 | NM_001042351 | Lim et al., 2005 | Non-Target | 0.038565 |
| 45 | hsa-miR-1 | NM_024792 | Lim et al., 2005 | Non-Target | 0.088619 |
| 46 | hsa-miR-1 | NM_172020 | Lim et al., 2005 | Non-Target | 0.002326 |
| 47 | hsa-miR-1 | NM_152450 | Lim et al., 2005 | Non-Target | Non-Target |
| 48 | hsa-miR-1 | NM_173505 | Lim et al., 2005 | Non-Target | Non-Target |
| 49 | hsa-miR-1 | NM_018290 | Lim et al., 2005 | Target | 0.041173 |
| 50 | hsa-miR-1 | NM_005345 | Lim et al., 2005 | Non-Target | Non-Target |
| 51 | hsa-miR-1 | NM_021913 | Lim et al., 2005 | Non-Target | 0.040004 |
| 52 | hsa-miR-1 | NM_022071 | Lim et al., 2005 | Non-Target | Non-Target |
| 53 | hsa-miR-1 | NM_001194 | Luo X et al., 2008 | Non-Target | Non-Target |
| 54 | hsa-miR-1 | NM_005477 | Luo X et al., 2008 | Non-Target | Non-Target |
| 55 | hsa-miR-1 | NM_022098 | Lim et al., 2005 | Non-Target | 0.074332 |
| 56 | hsa-miR-1 | NM_003271 | Lim et al., 2005 | Non-Target | Non-Target |
| 57 | hsa-miR-1 | NM_018330 | Lim et al., 2005 | Non-Target | 0.007044 |
| 58 | hsa-miR-1 | NM_015318 | Lim et al., 2005 | Non-Target | 0.05033 |
| 59 | hsa-miR-1 | NM_002999 | Lim et al., 2005 | Non-Target | 0.077922 |
| 60 | hsa-miR-1 | NM_016291 | Lim et al., 2005 | Non-Target | Non-Target |
| 61 | hsa-miR-1 | NM_016004 | Lim et al., 2005 | Non-Target | Non-Target |
| 62 | hsa-miR-124 | NM_152237 | Wang et al., 2006 | Target | 0.119233 |
| 63 | hsa-miR-124 | NM_021814 | Wang et al., 2006 | Target | 0.481316 |
| 64 | hsa-miR-124 | NM_001980 | Lim et al., 2005 | Target | 0.364604 |
| 65 | hsa-miR-124 | NM_002293 | Wang et al., 2006 | Target | 0.584593 |
| 66 | hsa-miR-124 | NM_002958 | Lim et al., 2005 | Target | 0.77393 |
| 67 | hsa-miR-124 | NM_003909 | Lim et al., 2005 | Target | 0.638204 |
| 68 | hsa-miR-124 | NM_003945 | Wang et al., 2006 | Target | 0.479647 |
| 69 | hsa-miR-124 | NM_004339 | Lim et al., 2005 | Target | 0.517053 |
| 70 | hsa-miR-124 | NM_004670 | Lim et al., 2005 | Target | 0.579115 |
| 71 | hsa-miR-124 | NM_004817 | Lim et al., 2005 | Target | Non-Target |
| 72 | hsa-miR-124 | NM_004945 | Lim et al., 2005 | Target | 0.209874 |
| 73 | hsa-miR-124 | NM_005327 | Lim et al., 2005 | Target | 0.145426 |
| 74 | hsa-miR-124 | NM_005498 | Lim et al., 2005 | Target | 0.189933 |
| 75 | hsa-miR-124 | NM_005730 | Lim et al., 2005 | Target | 0.530433 |
| 76 | hsa-miR-124 | NM_005754 | Lim et al., 2005 | Target | 0.366503 |
| 77 | hsa-miR-124 | NM_014447 | Lim et al., 2005 | Target | 0.358587 |
| 78 | hsa-miR-124 | NM_014481 | Lim et al., 2005 | Target | Non-Target |
| 79 | hsa-miR-124 | NM_014918 | Lim et al., 2005 | Target | 0.259692 |
| 80 | hsa-miR-124 | NM_015516 | Lim et al., 2005 | Target | 0.352866 |
| 81 | hsa-miR-124 | NM_015627 | Lim et al., 2005 | Target | 0.485755 |
| 82 | hsa-miR-124 | NM_018214 | Lim et al., 2005 | Target | 0.192839 |
| 83 | hsa-miR-124 | NM_019054 | Lim et al., 2005 | Target | 0.387621 |
| 84 | hsa-miR-124 | NM_020179 | Lim et al., 2005 | Target | 0.220403 |
| 85 | hsa-miR-124 | NM_022075 | Lim et al., 2005 | Target | 0.4939 |
| 86 | hsa-miR-124 | NM_024792 | Lim et al., 2005 | Target | 0.230419 |
| 87 | hsa-miR-124 | NM_024944 | Lim et al., 2005 | Target | 0.021372 |
| 88 | hsa-miR-124 | NM_031942 | Lim et al., 2005 | Target | 0.512005 |
| 89 | hsa-miR-124 | NM_152261 | Lim et al., 2005 | Target | 0.474502 |
| 90 | hsa-miR-124 | NM_153367 | Lim et al., 2005 | Target | 0.532582 |
| 91 | hsa-miR-124 | NM_172037 | Lim et al., 2005 | Target | 0.276354 |
| 92 | hsa-miR-124 | NM_172105 | Lim et al., 2005 | Target | 0.294143 |
| 93 | hsa-miR-124 | NM_000966 | Lim et al., 2005 | Target | 0.320649 |
| 94 | hsa-miR-124 | NM_001951 | Lim et al., 2005 | Target | 0.353437 |
| 95 | hsa-miR-124 | NM_002211 | Lim et al., 2005 | Target | 0.406344 |
| 96 | hsa-miR-124 | NM_002473 | Lim et al., 2005 | Target | 0.21558 |
| 97 | hsa-miR-124 | NM_002508 | Lim et al., 2005 | Target | 0.405516 |
| 98 | hsa-miR-124 | NM_002633 | Lim et al., 2005 | Target | 0.256832 |
| 99 | hsa-miR-124 | NM_002742 | Lim et al., 2005 | Target | 0.375708 |
| 100 | hsa-miR-124 | NM_002819 | Lim et al., 2005 | Target | 0.291838 |
| 101 | hsa-miR-124 | NM_002835 | Lim et al., 2005 | Target | 0.305053 |
| 102 | hsa-miR-124 | NM_003051 | Wang et al., 2006 | Target | 0.335992 |
| 103 | hsa-miR-124 | NM_003068 | Lim et al., 2005 | Target | 0.344378 |
| 104 | hsa-miR-124 | NM_003870 | Lim et al., 2005 | Target | 0.384439 |
| 105 | hsa-miR-124 | NM_004099 | Lim et al., 2005 | Target | 0.45919 |
| 106 | hsa-miR-124 | NM_004125 | Lim et al., 2005 | Target | 0.507951 |
| 107 | hsa-miR-124 | NM_004364 | Lim et al., 2005 | Target | 0.473408 |
| 108 | hsa-miR-124 | NM_004685 | Lim et al., 2005 | Target | 0.279328 |
| 109 | hsa-miR-124 | NM_004781 | Wang et al., 2006 | Target | 0.547375 |
| 110 | hsa-miR-124 | NM_004862 | Lim et al., 2005 | Target | 0.517344 |
| 111 | hsa-miR-124 | NM_005720 | Lim et al., 2005 | Target | 0.489072 |
| 112 | hsa-miR-124 | NM_005903 | Lim et al., 2005 | Target | 0.745767 |
| 113 | hsa-miR-124 | NM_006016 | Wang et al., 2006 | Target | 0.583058 |
| 114 | hsa-miR-124 | NM_006111 | Wang et al., 2006 | Target | 0.25989 |
| 115 | hsa-miR-124 | NM_006320 | Lim et al., 2005 | Target | 0.235669 |
| 116 | hsa-miR-124 | NM_006467 | Lim et al., 2005 | Target | 0.282524 |
| 117 | hsa-miR-124 | NM_006496 | Lim et al., 2005 | Target | 0.497903 |
| 118 | hsa-miR-124 | NM_014320 | Lim et al., 2005 | Target | 0.133029 |
| 119 | hsa-miR-124 | NM_014445 | Wang et al., 2006 | Target | 0.560064 |
| 120 | hsa-miR-124 | NM_014762 | Lim et al., 2005 | Target | 0.31256 |
| 121 | hsa-miR-124 | NM_016839 | Lim et al., 2005 | Target | 0.555641 |
| 122 | hsa-miR-124 | NM_018226 | Lim et al., 2005 | Target | 0.142017 |
| 123 | hsa-miR-124 | NM_020360 | Lim et al., 2005 | Target | 0.353625 |
| 124 | hsa-miR-124 | NM_021961 | Lim et al., 2005 | Target | 0.530463 |
| 125 | hsa-miR-124 | NM_022365 | Lim et al., 2005 | Target | 0.105028 |
| 126 | hsa-miR-124 | NM_032139 | Lim et al., 2005 | Target | 0.296646 |
| 127 | hsa-miR-124 | NM_032236 | Lim et al., 2005 | Target | 0.412418 |
| 128 | hsa-miR-124 | NM_145648 | Lim et al., 2005 | Target | 0.256232 |
| 129 | hsa-miR-124 | NM_152237 | Lim et al., 2005 | Target | 0.119233 |
| 130 | hsa-miR-124 | NM_172390 | Lim et al., 2005 | Target | 0.402723 |
| 131 | hsa-miR-124 | NM_000104 | Lim et al., 2005 | Target | 0.667811 |
| 132 | hsa-miR-124 | NM_000183 | Lim et al., 2005 | Target | 0.107334 |
| 133 | hsa-miR-124 | NM_000611 | Lim et al., 2005 | Target | 0.569291 |
| 134 | hsa-miR-124 | NM_001099678 | Lim et al., 2005 | Target | 0.590811 |
| 135 | hsa-miR-124 | NM_001259 | Lim et al., 2005 | Target | 0.61634 |
| 136 | hsa-miR-124 | NM_001921 | Lim et al., 2005 | Target | 0.49231 |
| 137 | hsa-miR-124 | NM_003060 | Lim et al., 2005 | Target | 0.232594 |
| 138 | hsa-miR-124 | NM_004239 | Lim et al., 2005 | Target | 0.428848 |
| 139 | hsa-miR-124 | NM_004422 | Lim et al., 2005 | Target | 0.434563 |
| 140 | hsa-miR-124 | NM_004815 | Lim et al., 2005 | Target | 0.661445 |
| 141 | hsa-miR-124 | NM_005397 | Lim et al., 2005 | Target | 0.442303 |
| 142 | hsa-miR-124 | NM_006410 | Lim et al., 2005 | Target | 0.133621 |
| 143 | hsa-miR-124 | NM_014170 | Lim et al., 2005 | Target | 0.380583 |
| 144 | hsa-miR-124 | NM_014300 | Lim et al., 2005 | Target | 0.284048 |
| 145 | hsa-miR-124 | NM_014397 | Lim et al., 2005 | Target | 0.348339 |
| 146 | hsa-miR-124 | NM_014452 | Lim et al., 2005 | Target | 0.500758 |
| 147 | hsa-miR-124 | NM_018370 | Lim et al., 2005 | Target | 0.581613 |
| 148 | hsa-miR-124 | NM_018719 | Lim et al., 2005 | Target | 0.200753 |
| 149 | hsa-miR-124 | NM_018845 | Lim et al., 2005 | Target | 0.266526 |
| 150 | hsa-miR-124 | NM_019027 | Lim et al., 2005 | Target | 0.568432 |
| 151 | hsa-miR-124 | NM_019895 | Lim et al., 2005 | Target | 0.239476 |
| 152 | hsa-miR-124 | NM_022152 | Lim et al., 2005 | Target | 0.233229 |
| 153 | hsa-miR-124 | NM_032156 | Lim et al., 2005 | Target | 0.220664 |
| 154 | hsa-miR-124 | NM_144578 | Lim et al., 2005 | Target | 0.769876 |
| 155 | hsa-miR-124 | NM_152792 | Lim et al., 2005 | Target | 0.287309 |
| 156 | hsa-miR-124 | NM_153186 | Lim et al., 2005 | Target | 0.147106 |
| 157 | hsa-miR-124 | NM_173607 | Lim et al., 2005 | Target | 0.440952 |
| 158 | hsa-miR-124 | NM_175866 | Lim et al., 2005 | Target | 0.185504 |
| 159 | hsa-miR-124 | NM_181722 | Lim et al., 2005 | Target | 0.24968 |
| 160 | hsa-miR-124 | NM_206894 | Lim et al., 2005 | Target | 0.019333 |
| 161 | hsa-miR-124 | NM_213636 | Lim et al., 2005 | Target | 0.29816 |
| 162 | hsa-miR-124 | NM_000075 | Lim et al., 2005 | Non-Target | 0.245005 |
| 163 | hsa-miR-124 | NM_002632 | Lim et al., 2005 | Non-Target | 0.152001 |
| 164 | hsa-miR-124 | NM_004402 | Lim et al., 2005 | Non-Target | 0.133044 |
| 165 | hsa-miR-124 | NM_030935 | Lim et al., 2005 | Non-Target | 0.171217 |
| 166 | hsa-miR-124 | NM_178507 | Lim et al., 2005 | Non-Target | 0.199347 |
| 167 | hsa-miR-124 | NM_001084 | Lim et al., 2005 | Non-Target | 0.116558 |
| 168 | hsa-miR-124 | NM_004706 | Lim et al., 2005 | Non-Target | 0.110155 |
| 169 | hsa-miR-124 | NM_006289 | Lim et al., 2005 | Non-Target | 0.087781 |
| 170 | hsa-miR-124 | NM_004710 | Lim et al., 2005 | Non-Target | 0.156768 |
| 171 | hsa-miR-124 | NM_145204 | Lim et al., 2005 | Non-Target | 0.127596 |
| 172 | hsa-miR-124 | NM_178439 | Lim et al., 2005 | Non-Target | 0.238446 |
| 173 | hsa-miR-17-5p | NM_006534 | Hossain et al., 2006, Nielsen et al., 2007 | Target | 0.67047 |
| 174 | hsa-miR-206 | NM_007124 | Rosenberg et al., 2006; Nielsen et al., 2007 | Target | 0.303744 |
| 175 | hsa-miR-206 | NM_007085 | Rosenberg et al., 2006; Nielsen et al., 2007 | Target | 0.183358 |
| 176 | hsa-miR-208a | NM_005121 | Nielsen et al., 2007 | Target | 0.287979 |
| 177 | hsa-miR-21 | NM_000366 | Nielsen et al., 2007 | Non-Target | Non-Target |
|  |  |  |  | Sensitivity = 77.96 % | Sensitivity = 89.83 % |
