# Supplementary material for: MultiMiTar: A Novel Multi Objective Optimization based miRNA-Target Prediction Method
Source: PLoS One. 2011 Sep 15;6(9):e24583. doi: 10.1371/journal.pone.0024583 (PMC3174180; doi:10.1371/journal.pone.0024583)
Supplement: Table S5 — Performance Comparison between MultiMiTar and TargetMiner based on 47 completely independent biologically validated negative test examples. (DOC) [file pone.0024583.s005.doc]

|  | **miRNA** | **mRNA** | **TargetMiner Prediction** | **MultiMiTar**  **Prediction** |
| --- | --- | --- | --- | --- |
| 1 | dme-miR-278 | NM_057426 | Non-Target | Non-Target |
| 2 | dme-miR-286 | NM_080709 | Non-Target | Non-Target |
| 3 | dme-miR-287 | NM_080265 | Non-Target | Non-Target |
| 4 | dme-miR-317 | NM_078846 | Non-Target | Non-Target |
| 5 | dme-miR-318 | NM_001038941 | Non-Target | Non-Target |
| 6 | hsa-let-7b | NM_001614 | Non-Target | Non-Target |
| 7 | hsa-let-7b | NM_001237 | Target | Target |
| 8 | hsa-miR-1 | NM_021109 | Non-Target | Non-Target |
| 9 | hsa-miR-1 | NM_001111285 | Non-Target | Non-Target |
| 10 | hsa-miR-103 | NM_005276 | Target | Target |
| 11 | hsa-miR-124 | NM_004781 | Target | Target |
| 12 | hsa-miR-124 | NM_001621 | Target | Target |
| 13 | hsa-miR-124 | NM_024551 | Target | Target |
| 14 | hsa-miR-124a | NM_138973 | Target | Target |
| 15 | hsa-miR-126 | NM_033102 | Non-Target | Non-Target |
| 16 | hsa-miR-128 | NM_003176 | Non-Target | Non-Target |
| 17 | hsa-miR-138 | NM_033102 | Non-Target | Non-Target |
| 18 | hsa-miR-141 | NM_006281 | Non-Target | Non-Target |
| 19 | hsa-miR-141 | NM_001730 | Non-Target | Non-Target |
| 20 | hsa-miR-145 | NM_014547 | Non-Target | Non-Target |
| 21 | hsa-miR-155 | NM_024900 | Non-Target | Non-Target |
| 22 | hsa-miR-15a | NM_138973 | Target | Target |
| 23 | hsa-miR-16 | NM_001241 | Target | Target |
| 24 | hsa-miR-19a | NM_019102 | Non-Target | Target |
| 25 | hsa-miR-19b | NM_138973 | Target | Target |
| 26 | hsa-miR-200a | NM_030751 | Non-Target | Non-Target |
| 27 | hsa-miR-29c | NM_054016 | Non-Target | Non-Target |
| 28 | hsa-miR-302a | NM_003182 | Non-Target | Non-Target |
| 29 | hsa-miR-375 | NM_130439 | Non-Target | Non-Target |
| 30 | hsa-miR-375 | NM_002196 | Non-Target | Non-Target |
| 31 | hsa-miR-429 | NM_030751 | Target | Non-Target |
| 32 | mmu-let-7b | NM_009075 | Non-Target | Non-Target |
| 33 | mmu-let-7b | NM_009609 | Non-Target | Non-Target |
| 34 | mmu-miR-1 | NM_021278 | Non-Target | Non-Target |
| 35 | mmu-miR-124 | NM_009498 | Target | Target |
| 36 | mmu-miR-124 | NM_197985 | Target | Target |
| 37 | mmu-miR-124 | NM_013464 | Target | Target |
| 38 | mmu-miR-141 | NM_009769 | Non-Target | Non-Target |
| 39 | mmu-miR-141 | NM_019635 | Non-Target | Non-Target |
| 40 | mmu-miR-141 | NM_025656 | Non-Target | Non-Target |
| 41 | mmu-miR-141 | NM_014795 | Non-Target | Non-Target |
| 42 | mmu-miR-16 | NM_028399 | Target | Target |
| 43 | mmu-miR-200a | NM_030751 | Non-Target | Non-Target |
| 44 | mmu-miR-215 | NM_014795 | Non-Target | Non-Target |
| 45 | mmu-miR-375 | NM_016889 | Non-Target | Non-Target |
| 46 | mmu-miR-375 | NM_001008542 | Non-Target | Non-Target |
| 47 | mmu-miR-429 | NM_030751 | Target | Non-Target |
|  |  |  | Specificity= 68.09 % | Specificity= 70.21 % |
