# Supplementary material for: MultiMiTar: A Novel Multi Objective Optimization based miRNA-Target Prediction Method
Source: PLoS One. 2011 Sep 15;6(9):e24583. doi: 10.1371/journal.pone.0024583 (PMC3174180; doi:10.1371/journal.pone.0024583)
Supplement: Table S6 — miRNA-targeting site context specific features (category-wise all the 90 features). (DOC) [file pone.0024583.s006.doc]

| Feat. No. | Feature Name |
| --- | --- |
| Category 1 | |
| 1 | Number of effective six mer seed matching sites |
| 2 | Number of local AU rich regions immediately flanking the effective six mer seed site |
| 3 | Number of additional Watson-Crick pairing associated with effective six mer |
| 4 | Number of effective seven mer m8 seed matching sites |
| 5 | Number of local AU rich regions immediately flanking the effective seven mer m8 seed site |
| 6 | Number of additional Watson-Crick pairing associated with effective seven mer m8 |
| 7 | Number of effective seven mer A1 seed matching sites |
| 8 | Number of local AU rich regions immediately flanking the effective seven mer A1 seed site |
| 9 | Number of additional Watson-Crick pairing associated with effective seven mer A1 |
| 10 | Number of eight mer effective seed matching sites |
| 11 | Number of local AU rich regions immediately flanking the eight mer effective seed site |
| 12 | Number of additional Watson-Crick pairing associated with effective eight mer |
| Frequency of Single nucleotide in seed matching site (Category 2) | |
| 13 | A’s frequency in effective seed matching site |
| 14 | U’s frequency in effective seed matching site |
| 15 | G’s frequency in effective seed matching site |
| 16 | C’s frequency in effective seed matching site |
|  | Frequency of Single nucleotide in seed matching out site (Category 3) |
| 17 | A’s frequency in effective seed matching out site |
| 18 | U’s frequency in effective seed matching out site |
| 19 | G’s frequency in effective seed matching out site |
| 20 | C’s frequency in effective seed matching out site |
| Frequency of Di - nucleotides frequency in seed matching site (Category 4) | |
| 21 | AA’s frequency in effective seed matching site |
| 22 | AU’s frequency in effective seed matching site |
| 23 | AG’s frequency in effective seed matching site |
| 24 | AC’s frequency in effective seed matching site |
| 25 | UA’s frequency in effective seed matching site |
| 26 | UU’s frequency in effective seed matching site |
| 27 | UG’s frequency in effective seed matching site |
| 28 | UC’s frequency in effective seed matching site |
| 29 | GA’s frequency in effective seed matching site |
| 30 | GU’s frequency in effective seed matching site |
| 31 | GG’s frequency in effective seed matching site |
| 32 | GC’s frequency in effective seed matching site |
| 33 | CA’s frequency in effective seed matching site |
| 34 | CU’s frequency in effective seed matching site |
| 35 | CG’s frequency in effective seed matching site |
| 36 | CC’s frequency in effective seed matching site |
| Frequency of Di - nucleotides in seed matching out site (Category 5) | |
| 37 | AA’s frequency in effective seed matching out site |
| 38 | AU’s frequency in effective seed matching out site |
| 39 | AG’s frequency in effective seed matching out site |
| 40 | AC’s frequency in effective seed matching out site |
| 41 | UA’s frequency in effective seed matching out site |
| 42 | UU’s frequency in effective seed matching out site |
| 43 | UG’s frequency in effective seed matching out site |
| 44 | UC’s frequency in effective seed matching out site |
| 45 | GA’s frequency in effective seed matching out site |
| 46 | GU’s frequency in effective seed matching out site |
| 47 | GG’s frequency in effective seed matching out site |
| 48 | GC’s frequency in effective seed matching out site |
| 49 | CA’s frequency in effective seed matching out site |
| 50 | CU’s frequency effective in seed matching out site |
| 51 | CG’s frequency in effective seed matching out site |
| 52 | CC’s frequency in effective seed matching out site |
| miRNA-mRNA base interaction features in seed region (Category 6) | |
| 53 | Frequency of AU base pair |
| 54 | Frequency of UA base pair |
| 55 | Frequency of UG base pair |
| 56 | Frequency of GC base pair |
| 57 | Frequency of GU base pair |
| 58 | Frequency of CG base pair |
| Two consecutive miRNA-mRNA base interaction features in seed region (Bi-Di-nucleotide base pairing) (Category 7) | |
| 59 | Frequency of AU-AU |
| 60 | Frequency of AU-UA |
| 61 | Frequency of AU-GC |
| 62 | Frequency of AU-CG |
| 63 | Frequency of AU-GU |
| 64 | Frequency of AU-UG |
| 65 | Frequency of UA-AU |
| 66 | Frequency of UA-UA |
| 67 | Frequency of UA-GC |
| 68 | Frequency of UA-CG |
| 69 | Frequency of UA-GU |
| 70 | Frequency of UA-UG |
| 71 | Frequency of GC-AU |
| 72 | Frequency of GC-UA |
| 73 | Frequency of GC-GC |
| 74 | Frequency of GC-CG |
| 75 | Frequency of GC-GU |
| 76 | Frequency of GC-UG |
| 77 | Frequency of CG-AU |
| 78 | Frequency of CG-UA |
| 79 | Frequency of CG-GC |
| 80 | Frequency of CG-CG |
| 81 | Frequency of CG-GU |
| 82 | Frequency of CG-UG |
| 83 | Frequency of GU-AU |
| 84 | Frequency of GU-UA |
| 85 | Frequency of GU-GC |
| 86 | Frequency of GU-CG |
| 87 | Frequency of UG-AU |
| 88 | Frequency of UG-UA |
| 89 | Frequency of UG-GC |
| 90 | Frequency of UG-CG |
