# Supplementary material for: MultiMiTar: A Novel Multi Objective Optimization based miRNA-Target Prediction Method
Source: PLoS One. 2011 Sep 15;6(9):e24583. doi: 10.1371/journal.pone.0024583 (PMC3174180; doi:10.1371/journal.pone.0024583)
Supplement: Table S7 — Performance of MultiMiTar and existing target prediction methods on the 28 experimentally verified Human miRNA-CDKN1A interactions. In the Table, 1 denotes target and 0 denotes non-target predicted by the respective algorithm. (DOC) [file pone.0024583.s007.doc]

| Sl.  No. | miRNA | MultiMiTar | TargetScan | TargetMiner | miRanda | mirtarget2 | PITA | PicTar | NBmiRTar | RNAhybrid | DIANA-microT 3.0 | microInspector |
| --- | --- | --- | --- | --- | --- | --- | --- | --- | --- | --- | --- | --- |
| 1 | miR-28-5p | 1 | 0 | 1 | 1 | 0 | 1 | 0 | 0 | 1 | 0 | 0 |
| 2 | miR-572 | 0 | 0 | 0 | 0 | 0 | 1 | 0 | 0 | 1 | 0 | 1 |
| 3 | miR-93 | 1 | 1 | 1 | 1 | 1 | 1 | 1 | 1 | 1 | 1 | 0 |
| 4 | miR-106b | 1 | 1 | 1 | 1 | 1 | 1 | 1 | 1 | 1 | 1 | 0 |
| 5 | miR-17-5p | 1 | 1 | 1 | 1 | 1 | 1 | 1 | 1 | 1 | 1 | 0 |
| 6 | miR-20a | 1 | 1 | 1 | 1 | 1 | 1 | 1 | 1 | 1 | 1 | 0 |
| 7 | miR-208a | 1 | 0 | 1 | 1 | 0 | 1 | 0 | 0 | 1 | 0 | 0 |
| 8 | miR-208b | 1 | 0 | 1 | 1 | 0 | 1 | 0 | 0 | 1 | 0 | 0 |
| 9 | miR-345 | 0 | 0 | 0 | 1 | 1 | 1 | 0 | 0 | 1 | 0 | 0 |
| 10 | miR-299-5p | 0 | 0 | 0 | 1 | 0 | 1 | 0 | 0 | 1 | 0 | 0 |
| 11 | miR-654-3p | 0 | 0 | 0 | 1 | 0 | 1 | 0 | 0 | 1 | 0 | 0 |
| 12 | miR-132 | 0 | 1 | 0 | 1 | 0 | 1 | 0 | 1 | 1 | 0 | 0 |
| 13 | miR-423-3p | 0 | 0 | 1 | 0 | 0 | 1 | 0 | 0 | 1 | 0 | 1 |
| 14 | miR-657 | 0 | 0 | 0 | 0 | 0 | 1 | 0 | 1 | 1 | 0 | 1 |
| 15 | miR-639 | 1 | 0 | 1 | 0 | 0 | 1 | 0 | 0 | 1 | 0 | 1 |
| 16 | miR-125a-5p | 0 | 0 | 0 | 0 | 0 | 0 | 0 | 0 | 1 | 0 | 0 |
| 17 | miR-372 | 1 | 0 | 1 | 0 | 0 | 1 | 1 | 1 | 1 | 1 | 0 |
| 18 | miR-515-3p | 1 | 0 | 1 | 0 | 0 | 1 | 0 | 0 | 1 | 0 | 1 |
| 19 | miR-519b-3p | 1 | 0 | 1 | 1 | 0 | 1 | 0 | 0 | 1 | 1 | 0 |
| 20 | miR-519d | 1 | 1 | 1 | 1 | 1 | 1 | 0 | 1 | 1 | 1 | 0 |
| 21 | miR-519e | 1 | 0 | 1 | 0 | 0 | 1 | 0 | 0 | 1 | 0 | 1 |
| 22 | miR-520a-3p | 1 | 0 | 1 | 0 | 0 | 1 | 0 | 0 | 1 | 1 | 0 |
| 23 | miR-520b | 1 | 0 | 1 | 0 | 0 | 1 | 0 | 1 | 1 | 1 | 0 |
| 24 | miR-520h | 1 | 0 | 1 | 1 | 1 | 1 | 0 | 0 | 1 | 1 | 0 |
| 25 | miR-298 | 1 | 0 | 1 | 1 | 0 | 1 | 0 | 1 | 1 | 0 | 1 |
| 26 | miR-106a | 1 | 1 | 1 | 1 | 1 | 1 | 0 | 1 | 1 | 0 | 0 |
| 27 | miR-20b | 1 | 1 | 1 | 1 | 1 | 1 | 1 | 1 | 1 | 1 | 0 |
| 28 | miR-363 | 0 | 0 | 0 | 0 | 0 | 1 | 0 | 1 | 1 | 0 | 0 |
