# Supplementary material for: MultiMiTar: A Novel Multi Objective Optimization based miRNA-Target Prediction Method
Source: PLoS One. 2011 Sep 15;6(9):e24583. doi: 10.1371/journal.pone.0024583 (PMC3174180; doi:10.1371/journal.pone.0024583)
Supplement: Table S8 — a. Performance of MultiMiTar on KRAS (NM_033360) - hsa-let-7 interactions without considering GU pairing at seed matching site. b. Performance of MultiMiTar on KRAS (NM_033360) - hsa-let-7 interactions with considering single GU pairing at seed matching site. (DOC) [file pone.0024583.s008.doc]

Table S8A.

| MiRNA | mRNA | 6mer (count) | 6mer (position) | 7mer-A1 (count) | 7mer-A1 (position) | 7mer-m8 (count) | 7mer-m8 (position) | 8mer (count) | 8mer (position) | Score |
| --- | --- | --- | --- | --- | --- | --- | --- | --- | --- | --- |
| hsa-let-7a | NM_033360 | 1 | 3272 | - | - | - | - | - | - | 0.028984 |
| hsa-let-7b | NM_033360 | 1 | 3272 | - | - | - | - | - | - | 0.028984 |
| hsa-let-7c | NM_033360 | 1 | 3272 | - | - | - | - | - | - | 0.028984 |
| hsa-let-7d | NM_033360 | 1 | 3272 | - | - | - | - | - | - | 0.028984 |
| hsa-let-7e | NM_033360 | 1 | 3272 | - | - | - | - | - | - | 0.028984 |
| hsa-let-7f | NM_033360 | 1 | 3272 | - | - | - | - | - | - | 0.028984 |
| hsa-let-7g | NM_033360 | 1 | 3272 | - | - | - | - | - | - | 0.028984 |
| hsa-let-7i | NM_033360 | 1 | 3272 | - | - | - | - | - | - | 0.028984 |

Table S8b.

| miRNA | mRNA | 6mer (count) | 6mer (position) | 7mer-A1 (count) | 7mer-A1 (position) | 7mer-m8 (count) | 7mer-m8 (position) | 8mer (count) | 8mer (position) | Score |
| --- | --- | --- | --- | --- | --- | --- | --- | --- | --- | --- |
| hsa-let-7a | NM_033360 | 8 | 4375 3878 3477 3393 3272 2930 411 214 | 3 | 3957 3306 256 | - | - | - | - | 0.634877 |
| hsa-let-7b | NM_033360 | 8 | 4375 3878 3477 3393 3272 2930 411 214 | 3 | 3957 3306 256 | - | - | - | - | 0.634877 |
| hsa-let-7c | NM_033360 | 8 | 4375 3878 3477 3393 3272 2930 411 214 | 3 | 3957 3306 256 | - | - | - | - | 0.634877 |
| hsa-let-7d | NM_033360 | 8 | 4375 3878 3477 3393 3272 2930 411 214 | 3 | 3957 3306 256 | - | - | - | - | 0.636442 |
| hsa-let-7e | NM_033360 | 8 | 4375 3878 3477 3393 3272 2930 411 214 | 3 | 3957 3306 256 | - | - | - | - | 0.634877 |
| hsa-let-7f | NM_033360 | 8 | 4375 3878 3477 3393 3272 2930 411 214 | 3 | 3957 3306 256 | - | - | - | - | 0.634877 |
| hsa-let-7g | NM_033360 | 8 | 4375 3878 3477 3393 3272 2930 411 214 | 3 | 3957 3306 256 | - | - | - | - | 0.634877 |
| hsa-let-7i | NM_033360 | 8 | 4375 3878 3477 3393 3272 2930 411 214 | 3 | 3957 3306 256 | - | - | - | - | 0.634877 |
